# Shifting stage‐specific constraints on productivity shape recovery potential for Yukon River Chinook salmon

**DOI:** 10.1002/eap.70229

**Published:** 2026-04-08

**Authors:** Lukas B. DeFilippo, Kathrine G. Howard, Curry J. Cunningham, Robert M. Suryan, Patrick D. Barry, James M. Murphy, Wesley A. Larson

**Affiliations:** ^1^ NOAA, Alaska Fisheries Science Center, Auke Bay Laboratories, Ecosystem Monitoring and Assessment Program Seattle Washington USA; ^2^ NOAA, Alaska Fisheries Science Center, Auke Bay Laboratories, Ecosystem Monitoring and Assessment Program Juneau Alaska USA; ^3^ University of Alaska Fairbanks, College of Fisheries and Ocean Sciences Juneau Alaska USA; ^4^ NOAA, Alaska Fisheries Science Center, Auke Bay Laboratories, Recruitment, Energetics, and Coastal Assessment Program Juneau Alaska USA; ^5^ NOAA, Alaska Fisheries Science Center, Auke Bay Laboratories, Genetics Program Juneau Alaska USA

**Keywords:** bycatch, Chinook salmon, integrated population model (IPM), life‐cycle model, marine heatwave, Yukon River

## Abstract

Identifying key life history periods in which population productivity is constrained represents a persistent challenge in conservation and natural resource management. For species with complex life cycles, such as Pacific salmon (*Oncorhynchus* spp.), population dynamics may be shaped by interactions between natural and anthropogenic impacts occurring across multiple habitats and life history stages. In such cases, a stage‐structured modeling approach is useful for identifying key life history periods and processes therein acting to drive realized abundance trends. Here, we develop an integrated life‐cycle model to explore stage‐specific constraints on population productivity and recovery potential for Yukon River Chinook salmon. The Yukon River has historically supported one of the largest stock complexes of Chinook salmon in the world, forming the basis of important fisheries that are vital to the well‐being of communities in this region. However, returns of Chinook salmon to the Yukon River have declined substantially, prompting conservation concerns and limitations on harvest opportunities. Our results point to periods of low juvenile recruitment as likely contributors to declining abundance levels over the past two decades, supporting previous studies implicating factors operating in the early (i.e., spawner‐to‐juvenile) life history stages. However, we find that elevated natural mortality in later, post‐juvenile life history stages has increasingly limited population productivity and recovery potential in recent years following a protracted marine heatwave period. Collectively, our results emphasize how shifting conditions can induce novel stage‐specific survival bottlenecks in species with complex life cycles, with important implications for conservation and management outcomes.

## INTRODUCTION

A foundational objective in conservation and natural resource management is to understand the drivers of variability in exploited populations (Hjort, 1914). Clear relationships between population dynamics and environmental patterns are elusive, and it can be difficult to disentangle the impacts of natural fluctuations versus harvest on observed abundance trends (Walters & Martell, 2004). Consequently, it is often challenging to diagnose the origins of population declines, and by extension, navigate pathways to recovery (Hilborn & Litzinger, 2009; Myers et al., 1997). These challenges are compounded for species with complex life histories, such as Pacific salmon (*Oncorhynchus* spp.), where the scope of potential natural and human impacts shifts across the life cycle (Bull et al., 2022; Crozier et al., 2021; Crozier & Zabel, 2006). In such cases, a stage‐structured examination of the population can be useful for identifying cryptic variation in life history processes underlying realized declines in abundance, and informing prospects for recovery (Kendall et al., 2023; Olmos et al., 2020).

Life‐cycle models are valuable tools for characterizing stage‐specific impacts on populations and evaluating potential responses to management actions and future conditions (Crozier et al., 2008; Wainwright & Weitkamp, 2013). Given the limited data typically available to estimate biological processes at each life history stage, early modeling efforts often relied on preexisting estimates and disparate data sources to inform parameter values (e.g., Moussalli & Hilborn, 1986; Scheuerell et al., 2006). More recently, integrated approaches to life‐cycle modeling have been developed that leverage all available information to directly estimate parameters via a joint likelihood (Buhle et al., 2018). Such integrated population models (IPMs) represent biological quantities as latent, unobserved states that are informed by multiple data sources with associated observation errors (e.g., DeFilippo et al., 2021). Consequently, IPMs can resolve important biological processes that may not be identifiable from isolated data sources, making them a useful tool for characterizing stage‐specific constraints on population productivity and informing conservation actions (Scheuerell et al., 2021; Sorel et al., 2021). Moreover, IPMs can also provide a basis for simulation‐testing population responses to alternative scenarios in a manner that propagates uncertainty in the data and parameter estimates.

Chinook salmon (*Oncorhynchus tshawytscha*) have exhibited widespread declines in abundance throughout their distribution (Atlas et al., 2023; Dorner et al., 2018; Ohlberger et al., 2016). For stocks that occupy the southern edge of the species' range or rely on heavily impacted watersheds, depressed productivity may be readily attributable to warming and/or habitat degradation (Crozier et al., 2008, 2021; Lindley et al., 2009; Sabal et al., 2016; Yates et al., 2008). Conversely, the causes of dwindling Chinook salmon stocks within the northern portion of their range, in relatively intact watersheds, are less clear (Schindler et al., 2013). The Yukon River, which spans the US state of Alaska and Canada's British Columbia province and Yukon Territory, has historically supported a large stock complex of Chinook salmon that formed the basis of important subsistence, commercial, and recreational fisheries (Evenson et al., 2009). However, Yukon River Chinook salmon run sizes have diminished substantially (by ~90% between 1981 and 2023), resulting in federal fishery disaster declarations, failure to meet treaty‐mandated fish passage targets across the US–Canada border, and curtailment of harvest opportunities (ADF&G, 2024; JTC, 2025; Loring & Gerlach, 2010; Schindler et al., 2013). Given the immense cultural, economic, and nutritional importance of Chinook salmon for communities associated with the Yukon River, understanding the basis of these declines and navigating pathways to recovery are conservation and management priorities.

In this study, we developed an IPM that incorporates multiple data sources to model the full life cycle of Yukon River Chinook salmon. We focus specifically on upper (Canada‐origin) Yukon River Chinook salmon here due to the genetic distinguishability of this run component and the availability of stock‐specific data necessary for model estimation. The objectives of our analysis are first to quantify key stage‐specific processes that have potentially contributed to declining abundance levels, with a focus on juvenile recruitment, post‐juvenile natural mortality, bycatch, and harvest removals. Next, we implement a retrospective simulation analysis to compare the relative influence of these processes in driving past declines by exploring outcomes under alternative recruitment, natural mortality, harvest, and bycatch removal histories. Finally, we leverage forward projections to identify limiting factors to future recovery outcomes. By characterizing stage‐specific constraints on population productivity and recovery potential, this study supports ongoing research and management efforts aimed at rebuilding Yukon River Chinook salmon and promoting the well‐being of the communities that they support.

## METHODS

### Yukon River Chinook salmon life history and historical abundance trends

**FIGURE 1:**
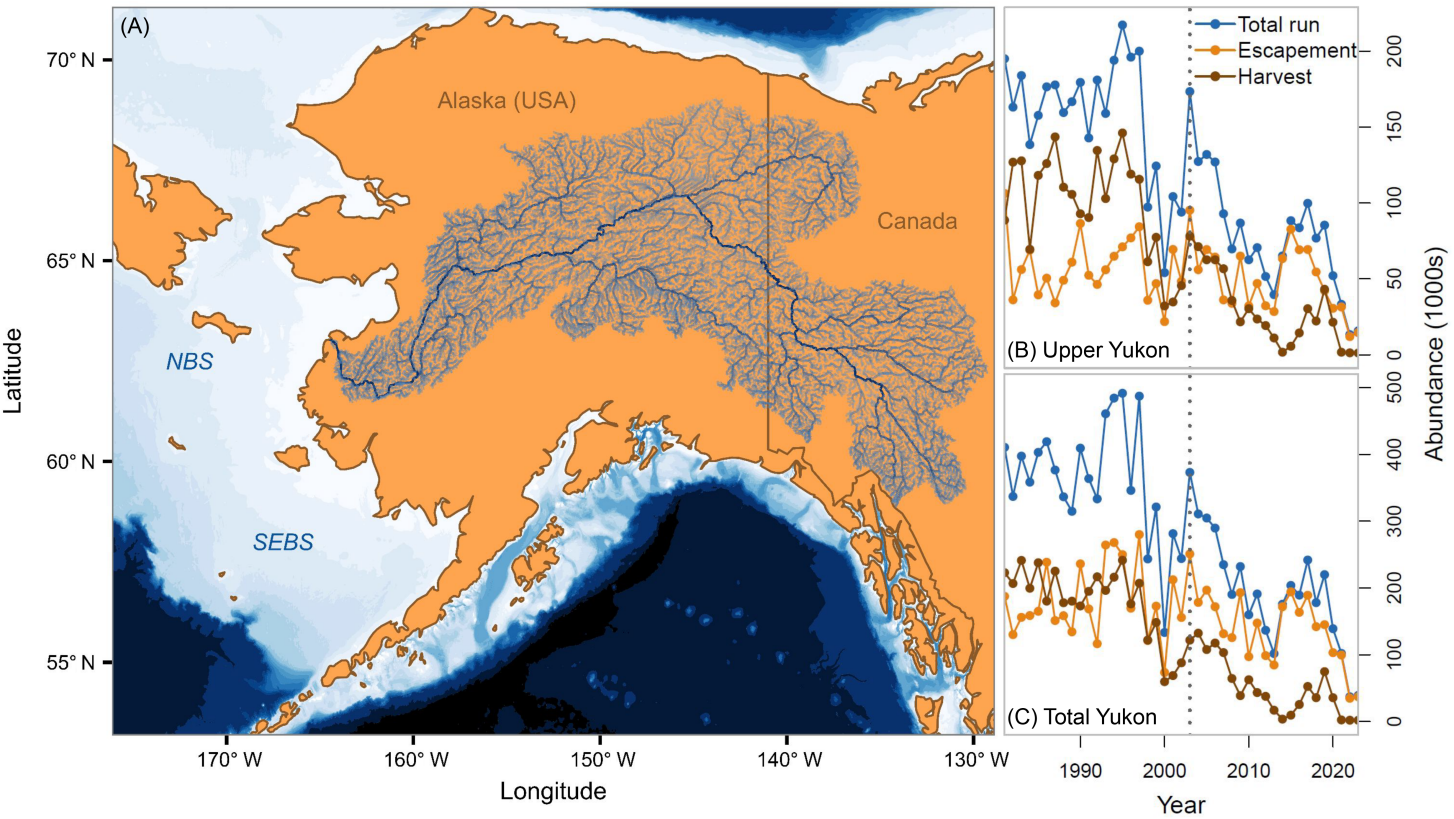
Map of the Yukon River watershed and Bering Sea shelf, and historical patterns of Yukon River Chinook salmon abundance. Panel (A) shows the Yukon River basin and the northern (NBS) and southeastern (SEBS) Bering Sea shelf. Panels (B) and (C) show the historical abundance of upper (Canada‐origin) and total Yukon River Chinook salmon, respectively, based on output from the Yukon River run reconstruction. In panels (B) and (C), total run size is shown in blue, escapement abundance is shown in orange, and harvest is shown in brown.

Yukon River Chinook salmon exhibit a “stream‐type” life history in which fish generally spend a year rearing in freshwater after overwintering in the gravel as eggs and between 2 and 5 years (with most individuals spending 3 or 4 years) in the ocean (Gilbert, 1922; Healey, 1991; Riddell et al., 2018). Individuals typically migrate from freshwater to the nearshore marine environment of the Northern Bering Sea (NBS) shelf (Figure 1A) in June following ice‐breakup, but outmigration can extend into August (Bradford et al., 2001). After a brief residency on the NBS shelf, it is thought that Yukon River Chinook salmon begin migrating offshore by their first ocean winter, and at later ocean ages may spend summers in the central and/or northwestern Bering Sea and winters in the southeastern Bering Sea (Larson et al., 2013; Myers et al., 2001, 2009, 2010) before returning to freshwater to spawn. These marine migration patterns overlap to some extent with the eastern Bering Sea (EBS) walleye pollock (*Gadus chalcogrammus*) (henceforth pollock) fishery (DeFilippo et al., 2025), resulting in bycatch of Yukon River Chinook salmon (Guthrie et al., 2022).

Historically, the Yukon River's Chinook salmon runs were among the largest in the world, with annual averages of nearly 400,000 fish river‐wide, and roughly 175,000 for the upper (Canada) portion of the Yukon River from 1981 to 1997 (Figure 1B,C). However, both Canada‐origin and total Yukon River Chinook salmon abundance fell sharply from 1998 to 2002, followed by a substantial uptick in 2003 (Figure 1B,C). Run sizes exhibited a steady downturn after 2003, punctuated by a partial recovery in the mid‐2010s and a precipitous drop between 2019 and 2023 (Figure 1B,C). Recent (2023) run sizes were <40,000 and <16,000 fish for the total and upper Yukon River, respectively, representing an order of magnitude reduction from historical abundance levels (Figure 1B,C).

### Model overview

**FIGURE 2:**
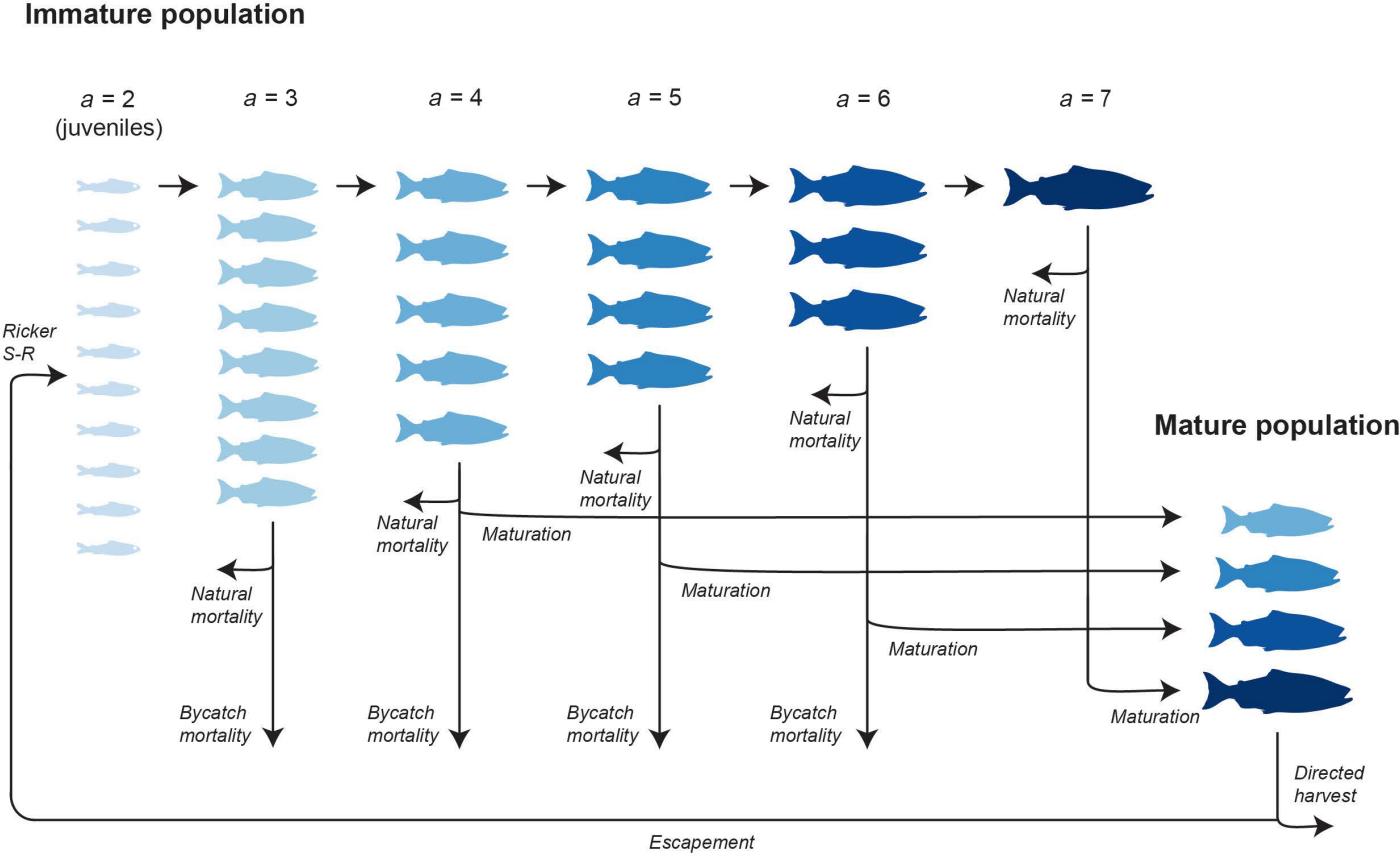
Model design and stage‐specific life cycle processes. Individuals are recruited as age‐2 (*a* = 2)/ocean age‐0 juveniles at the point when they are encountered by the Northern Bering Sea Ecosystem and Surface Trawl (NBEST) survey. At subsequent ages (*a* = 3–7), immature fish may be removed due to bycatch or natural mortality, or from reaching maturity and returning to freshwater to spawn. Mature individuals are subject to directed harvest, with those that survive comprising the spawning escapement that gives rise to future juvenile cohorts via a Ricker spawner‐to‐juvenile stock‐recruit (S‐R) function. Image credit: Juliana Cornett.

Our IPM incorporates estimates of juvenile marine abundance generated from cooperative US National Oceanic and Atmospheric Administration (NOAA) and Alaska Department of Fish and Game (ADF&G) Northern Bering Sea Ecosystem and Surface Trawl (NBEST) surveys (Murphy et al., 2017, 2023), bycatch abundance and age/stock composition information collected from the EBS pollock fishery (Cahalan et al., 2014; Guthrie et al., 2022), and run reconstruction‐based estimates of adult harvest and escapement at‐age information (Connors et al., 2023) to model the full life cycle of Yukon River Chinook salmon via a Bayesian estimation approach. In the population dynamics component of the IPM, individuals are recruited as age‐2 (ocean age‐0) juveniles encountered by the NBEST survey (Figure 2). Thus, variation in juvenile recruitment over time reflects a combination of reproductive output, freshwater mortality, and early (i.e., ~June to August of the outmigration year) marine mortality that occurs prior to the NBEST survey. After recruiting to the juvenile stage, individuals may subsequently be removed from the immature population due to factors such as bycatch mortality, post‐juvenile natural mortality (i.e., natural mortality that occurs after the point in the life cycle at which juveniles are encountered by the NBEST survey), or reaching maturity and returning to freshwater (Figure 2). Mature fish may be subject to terminal commercial, sport, personal use, and domestic fisheries targeting Yukon River Chinook salmon (henceforth “harvest”), with those that are not harvested comprising the spawning escapement that produces subsequent juvenile year‐classes (Figure 2). Thus, population dynamics in the IPM are governed by variation in inputs to the stock via juvenile recruitment, and removals via bycatch, natural mortality, and harvest. The temporal scope of our study (constrained by the earliest availability of juvenile data) extends from 2003 to 2023, and thus does not include historical years of higher Yukon River Chinook salmon abundance. However, our study does encompass the years in which the most acute declines in run sizes have occurred (Figure 1B,C).

### Model inputs

Abundance information for marine juvenile (total age‐2/ocean age‐0) Yukon River Chinook salmon was based on data collected by the NBEST survey (Murphy et al., 2017, 2023). Surveys have occurred annually from late August to late September since 2003, excluding 2008 and 2020; additionally, data from 2005 are less reliable due to variation in the survey sampling grid employed and were omitted from our analysis. The survey typically samples ~40 stations between 60° and 66.5° N and east of 171° W, with additional stations north of the Bering Strait included in some years. Sampling is conducted using a Cantrawl Pacific Ltd. 400/601 surface trawl with a typical tow duration of 30 min, a horizontal net opening of 40–60 m, a vertical net opening of 15–20 m, and a codend liner with 12‐mm mesh. Area swept (in square kilometers) is calculated as the product of haversine tow distance (measured using GPS positions for the beginning and end points of each tow), and the horizontal opening of the net as measured by a Simrad FS70 net sounder. Fish catch is enumerated by species and divided by area swept to calculate catch per unit effort (CPUE). CPUE is adjusted by mixed‐layer depth and expanded to the total survey area to produce an estimate of abundance, and juvenile Chinook salmon are assigned to genetic reporting groups (including the Canada‐origin Yukon River stock unit considered in this study) using mixed stock analysis. Complete details on sampling, abundance estimation, and genetic stock identification protocols can be found in Howard et al. (2019, 2020) and Murphy et al. (2017, 2023).

Data on bycatch of Chinook salmon in the EBS pollock fishery used in this study were collected through the US National Marine Fisheries Service (NMFS) observer sampling and catch accounting systems (Cahalan et al., 2014). Since 2011, the EBS pollock fleet has been subject to 100% fishery observer coverage with full census counts of all salmon caught, and paired genetic and scale samples collected from 1 in 10 fish. Prior to 2011, bycatch abundance was estimated by extrapolating the observed ratios of Chinook salmon bycatch to pollock catch from monitored vessels to the rest of the fleet (Ianelli & Stram, 2015). Age‐specific genetic stock composition estimates (including proportions‐at‐age assigned to the Canada‐origin Yukon River reporting group) were produced for the age classes frequently encountered in the bycatch (ages 3–6) by the NOAA Auke Bay Laboratories Genetics Program using a Bayesian conditional genetic stock identification model (Moran & Anderson, 2019), following the same methods used to estimate stock group contributions to the overall bycatch (Guthrie III et al., 2022).

Adult harvest and escapement at‐age information used in this study were produced from a run reconstruction that uses a Bayesian semi‐integrated state‐space framework fitted to in‐river run size (e.g., sonar, mark–recapture), harvest and tributary escapement abundance, and stock and age composition data (Connors et al., 2023). While estimates of run size are informed by abundance and stock composition data from the lower river (near the river mouth), natural mortality of adult spawners may occur during upriver spawning migrations (e.g., von‐Biela et al., 2022). For Canada‐origin Yukon River Chinook salmon, the run reconstruction ascribes greater certainty to abundance information for this stock generated from the upper river, near the US–Canada border, than from the lower river. Consequently, discrepancies between upper versus lower river abundance (e.g., due to unobserved en route mortality) may be aggregated with in‐river harvest estimates to some extent, and/or cause the run reconstruction's estimate of run size for the Canada‐origin stock to be reduced (i.e., to more closely resemble the upper river abundance estimates). Thus, with respect to the IPM, in‐river adult mortality may, to varying extents, manifest as post‐juvenile natural mortality and/or in‐river harvest.

### Model structure

#### Population dynamics

The abundance of juvenile (total age‐2/ocean age‐0) Canada‐origin Yukon River Chinook salmon ($N_{y,a=2}$) in year y was specified as a Ricker spawner‐recruit function (Ricker, 1954) of the escapement $\left(S\right)$ in the brood year (y−2) from which those juveniles originated:

(1) $$\mathrm{Log}\left(N_{y,a=2}\right)=\mathrm{Log}\left(S_{y-2}\right)+\mathrm{Log}\left(\alpha \right)-\beta S_{y-2}+{\varepsilon_{R}}_{y}$$

$${\varepsilon_{R}}_{y}\sim \text{Normal}\left(0,\sigma_{R}\right),$$

where α is the Ricker spawner‐to‐juvenile productivity parameter, β is the (inverse) capacity term, $\varepsilon_{R_{y}}$ is the recruitment error in year y, and $\sigma_{R}$ is the recruitment error SD.

For subsequent ages (a>2), the abundance of immature fish in year y was specified as:

(2) $$N_{y,a>2}=N_{y-1,a-1}\left(e^{-\left({F_{B}}_{y}{s_{B}}_{a}+M_{y}v_{a}\right)}\right)\left(1-\theta_{y_{c},a}\right),$$

where $N_{y-1,a-1}$ is the abundance of the previous age class in the previous year, ${F_{B}}_{y}$ is the fully selected bycatch mortality rate in year y, ${s_{B}}_{a}$ is the bycatch selectivity‐at‐age, $M_{y}$ is the post‐juvenile natural mortality rate in year y, and $v_{a}$ represents age‐specific vulnerability to natural mortality. Post‐juvenile natural mortality was assumed to follow a random walk in log space, with annual process errors (${\varepsilon_{M}}_{y}$) drawn from a normal distribution with a SD of $\sigma_{M}$:

(3) $$\;\mathrm{Log}\left(M_{y}\right)=\left\{\begin{array}{c} M_{\text{init}}\sim \text{Normal}\left(0,5\right),y=1\\ \mathrm{Log}\left(M_{y-1}\right)+{\varepsilon_{M}}_{y},y>1 \end{array}\right.$$

$${\varepsilon_{M}}_{y}\sim \text{Normal}\;\left(0,\sigma_{M}\right).$$

Similarly, the bycatch mortality rate was specified as a random walk process:

(4) $$\;\mathrm{Log}\left({F_{B}}_{y}\right)=\left\{\begin{array}{c} {F_{B}}_{\text{init}}\sim \text{Normal}\left(0,7.5\right),y=1\\ \mathrm{Log}\left({F_{B}}_{y-1}\right)+{\varepsilon_{B}}_{y},\;y>1 \end{array}\right.$$

$${\varepsilon_{B}}_{y}\sim \text{Normal}\left(0,\sigma_{B}\right).$$

Bycatch selectivity‐at‐age was assumed to be time‐invariant and was freely estimated. Vulnerability‐at‐age to natural mortality ($v_{a}$) was fixed at 1 for age‐3 individuals but estimated for subsequent ages such that vulnerability was assumed to be maximized at the youngest post‐juvenile age class (age‐3), but could decline for older ages.

Of the surviving immature fish, a portion may reach maturity and return to freshwater to spawn, where $\theta_{y_{c},a}$ represents the probability of maturing at age a for individuals from a given juvenile year‐class ($y_{c}=y-\left(a-2\right)$) such that fish from the same cohort were assumed to experience shared maturation probabilities‐at‐age. Age‐specific maturation probabilities ($\theta_{y_{c},a}$) were specified via a modified logistic function (Cunningham et al., 2018) that constrains individuals to reach maturity by age‐7, consistent with the observed age composition of Yukon River Chinook salmon:

(5) $$\;\theta_{y_{c},a}=\frac{1}{1+e^{\lambda_{y_{c}}\left(7-a\right)+\log\left(1/0.99-1\right)}},$$

where $\lambda_{y_{c}}$ controls the maturation schedule and was allowed to vary over time based on evidence of temporal shifts in Yukon River Chinook salmon age structure (Ohlberger et al., 2020). Variation in the maturation schedule term over time was assumed to follow a random walk in log space:

(6) $$\;\mathrm{Log}\left(\lambda_{y_{c}}\right)=\left\{\begin{array}{c} \lambda_{\text{init}}\sim \text{Normal}\left(1,1\right),y_{c}=1\\ \mathrm{Log}\left(\lambda_{y_{c}-1}\right)+{\varepsilon_{\lambda }}_{y_{c}},\;y_{c}>1 \end{array}\right.$$

$${\varepsilon_{\lambda }}_{y_{c}}\sim \text{Normal}\left(0,\sigma_{\lambda }\right),$$

where $\varepsilon_{{\lambda_{y}}_{c}}$ are process errors in the temporal evolution of the maturation schedule with SD $\sigma_{\lambda }$.

The number of Chinook salmon reaching maturity in a given year ($A_{y,a}$) was specified as:

(7) $$A_{y,a}=N_{y-1,a-1}\left(e^{-\left({F_{B}}_{y}{s_{B}}_{a}+M_{y}v_{a}\right)}\right)\left(\theta_{y_{c},a}\right),$$

with a portion of these mature fish subject to harvest ($H_{y,a}$):

(8) $$\;H_{y,a}=A_{y,a}\left(1-e^{-{F_{T}}_{y}{s_{T}}_{a}}\right),$$

where ${F_{T}}_{y}$ is the fully selected harvest mortality rate in year y, and ${s_{T}}_{a}$ are the harvest selectivity‐at‐age terms, which were freely estimated and time‐invariant. Harvest mortality was assumed to follow a random walk process over time:

(9) $$\;\mathrm{Log}\left({F_{T}}_{y}\right)=\left\{\begin{array}{c} {F_{T}}_{\text{init}}\sim \text{Normal}\left(0,5\right),y=1\\ \mathrm{Log}\left({F_{T}}_{y-1}\right)+{\varepsilon_{T}}_{y},\;y>1 \end{array}\right.$$

$${\varepsilon_{T}}_{y}\sim \text{Normal}\left(0,\sigma_{T}\right).$$

The spawning escapement‐at‐age in year y ($S_{y,a}$) was specified as the mature abundance minus those that were harvested:

(10) $$\;S_{y,a}=A_{y,a}-H_{y,a}.$$

The total annual spawning escapement $\left(S_{y}\right)$ was specified as the sum across all mature age classes (ages 4–7) in the escapement:

(11) $$\;S_{y}=\sum_{a=4}^{a=7}S_{y,a},$$

which was used recursively in the Ricker spawner‐recruit function in Equation (1).

#### Likelihoods

The data available for conditioning the population dynamics model described above include (1) abundance data for juvenile Canada‐origin Yukon River Chinook salmon generated from the NBEST survey; (2) Chinook salmon bycatch abundance, age composition, and age‐specific stock composition data from the EBS pollock fishery; and (3) run reconstruction‐derived adult harvest and escapement abundance and age composition information.

Juvenile abundance was assumed to follow a lognormal likelihood:

(12) $$\;J_{y}\sim \text{Lognormal}\left(N_{y,a=2},\sigma_{J}\right),$$

where $J_{y}$ is the abundance of juvenile Canada‐origin Yukon River Chinook salmon in year y generated from the NBEST survey, $N_{y,a=2}$ is the model‐predicted juvenile abundance, and $\sigma_{J}$ is the observation error SD, which was fixed at 0.25.

Escapement abundance was assumed to follow a lognormal likelihood as well:

(13) $$\;E_{y}\sim \text{Lognormal}\left(\sum_{a=4}^{a=7}S_{y,a},\sigma_{E}\right),$$

where $E_{y}$ is the total Canada‐origin escapement in year y generated from the run reconstruction, $S_{y,a}$ is the model‐predicted escapement abundance, and $\sigma_{E}$ is the observation error SD, which was fixed at 0.1.

Similarly, terminal harvest abundance was assumed to follow a lognormal likelihood:

(14) $$\;C_{y}\sim \text{Lognormal}\left(\sum_{a=4}^{a=7}H_{y,a},\sigma_{C}\right),$$

where $C_{y}$ is the total catch of Canada‐origin Yukon River Chinook salmon in year y generated from the run reconstruction, $H_{y,a}$ is the model‐predicted harvest abundance, and $\sigma_{C}$ is the observation error SD, which was fixed at 0.15.

The age composition of the escapement was assumed to follow a multinomial likelihood:

(15) $$\;{\pi_{S}}_{y,a}=\frac{S_{y,a}}{\sum_{a=4}^{a=7}S_{y,a}}$$

$${q_{E}}_{y,a}{n_{\mathrm{eff}}}_{E}\sim \text{Multinomial}\left({\pi_{S}}_{y,a}\right),$$

where ${\pi_{S}}_{y,a}$ are the model‐predicted age composition proportions, ${q_{E}}_{y,a}$ represents the run reconstruction‐derived age composition of the Canada‐origin escapement, and ${n_{\mathrm{eff}}}_{E}$ is the effective sample size of the escapement age composition, which was fixed at 100.

Similarly, the age composition of the harvest was assumed to follow a multinomial likelihood:

(16) $$\;{\pi_{H}}_{y,a}=\frac{H_{y,a}}{\sum_{a=4}^{a=7}H_{y,a}}$$

$${q_{C}}_{y,a}{n_{\mathrm{eff}}}_{C}\sim \text{Multinomial}\left({\pi_{H}}_{y,a}\right),$$

where ${\pi_{H}}_{y,a}$ are the model‐predicted age composition proportions, ${q_{C}}_{y,a}$ represents the age composition of the Canada‐origin Yukon River Chinook salmon harvest generated from the run reconstruction, and ${n_{\mathrm{eff}}}_{C}$ is the effective sample size of the harvest age composition, which was fixed at 100.

There are three data types that provide information on bycatch of Canada‐origin Yukon River Chinook salmon in the EBS pollock fishery: (1) the total abundance of the Chinook salmon bycatch, (2) the age composition of the total Chinook salmon bycatch, and (3) the age‐specific proportions of the bycatch genetically assigned to the Canada‐origin Yukon River reporting group each year. To scale the model‐predicted Canada‐origin Yukon River bycatch to the level of the total Chinook salmon bycatch, for which the age composition and abundance data exist, the model‐predicted Canada‐origin bycatch abundance‐at‐age $\left(B_{y,a}\right)$ was calculated as:

(17) $$B_{y,a}=\left(\frac{{F_{B}}_{y}{s_{B}}_{a}}{{F_{B}}_{y}{s_{B}}_{a}+M_{y}v_{a}}\right)\left(N_{y-1,a-1}\left(1-e^{-\left({F_{B}}_{y}{s_{B}}_{a}+M_{y}v_{a}\right)}\right)\right).$$

These estimates were then scaled to the level of the aggregate EBS Chinook salmon bycatch abundance‐at‐age (${B_{A}}_{y,a}$) based on the estimated proportions of each age class genetically assigned to the Canada‐origin Yukon River reporting group:

(18) $${B_{A}}_{y,a}=\frac{B_{y,a}}{\vartheta_{y,a}},$$

where $\vartheta_{y,a}$ is the model‐predicted proportion of the age a Chinook salmon bycatch composed of Canada‐origin Yukon River fish.

Model‐predicted bycatch stock composition proportions‐at‐age were hierarchically distributed among years to inform time periods with missing genetic stock assignment data:

(19) $$\text{Logit}\left(\vartheta_{y,a}\right)\sim \text{Normal}\left({\mu_{G}}_{a},{\sigma_{G}}_{a}\right),$$

where ${\mu_{G}}_{a}$ and $\;{\sigma_{G}}_{a}$ are the means and SDs of the among‐year distributions of the logit proportions‐at‐age belonging to the Canada‐origin Yukon River reporting group. The age‐specific bycatch stock composition data were assumed to follow a binomial likelihood:

(20) $$x_{y,a}\sim \text{Binomial}\left(n_{y,a},\vartheta_{y,a}\right),$$

where $x_{y,a}$ is the number of genetic samples of age a assigned to the Canada‐origin Yukon River reporting group in year y, and $n_{y,a}$ is the total sample size of age a Chinook salmon in the bycatch available for genotyping in year y.

The age composition proportions of the total EBS Chinook salmon bycatch were then calculated as:

(21) $${\pi_{B}}_{y,a}=\frac{{B_{A}}_{y,a}}{\sum_{a=3}^{a=6}{B_{A}}_{y,a}}.$$

The aggregate bycatch age composition data were assumed to follow a multinomial likelihood:

(22) $$\;{q_{B}}_{y,a}{n_{B}}_{y}\sim \text{Multinomial}\left({\pi_{B}}_{y,a}\right),$$

where ${q_{B}}_{y,a}$ are the observed age composition proportions for the EBS pollock fishery's Chinook salmon bycatch in year y, and ${n_{B}}_{y}$ are the total number of scale samples collected for aging.

The aggregate abundance of Chinook salmon bycatch was assumed to follow a lognormal likelihood:

(23) $$b_{y}\sim \text{Lognormal}\left(\sum_{a=3}^{a=6}{B_{A}}_{y,a},\sigma_{B}\right),$$

where $b_{y}$ is the total observed count of Chinook salmon bycatch and $\sigma_{B}$ is the associated observation error term, which was fixed at 0.1.

### Model estimation and validation

Model estimation was conducted in a Bayesian framework via Hamiltonian Monte Carlo (HMC) No‐U‐turn sampling (NUTS) through the Stan model building software implemented in R (4.3.2) (R Core team, 2023) using the Rstan (2.32.7) package (Stan Development Team, 2025). Posterior sampling occurred across five HMC chains with lengths of 15,000 iterations each. The first 5000 samples of each chain were discarded as a “warmup,” and each subsequent sample was saved to construct the posterior distribution. Convergence was assessed using the Gelman–Rubin diagnostic (Gelman & Rubin, 1992) $\left(\hat{R}\leq 1.01\right)$ and effective number of samples (≥1000). Posterior sampling was monitored for divergent transitions and low Bayesian fraction of missing information (BFMI), neither of which was present. Supporting information and analyses from the model are presented in Appendix S1. Model goodness of fit was assessed by comparing model predictions to the data (Appendix S2), and model performance was validated by posterior predictive checks (Appendix S3) and simulation testing (Appendix S4). We evaluated alternative plausible values of observation error terms and multinomial effective sample sizes, which did not qualitatively alter model outcomes. Prior distributions for all model parameters can be found in Table 1.

**TABLE 1:** Model prior distributions.

Term	Description	Prior
$\mathrm{Log}\left(\alpha \right)$	Log Ricker productivity	Normal(0,12.5)
β	Ricker (inverse) capacity	Normal(0,5)[0,∞]
$\sigma_{R}$	Recruitment error SD	Normal(0,5) [0,∞]
${s_{B}}_{a}$	Bycatch selectivity‐at‐age	Normal(0,1) [0,1]
$v_{a}$	Vulnerability‐at‐age to natural mortality	Normal(0,1) [0.5,1]
${s_{T}}_{a}$	Harvest selectivity‐at‐age	Normal(0,1) [0,1]
$\sigma_{M}$	Log natural mortality process error SD	Normal(0,5)[0,∞]
$\sigma_{B}$	Log bycatch mortality process error SD	Normal(0,5)[0,∞]
$\sigma_{\lambda }$	Log maturation schedule process error SD	Normal(0,5)[0,∞]
$\sigma_{T}$	Log harvest mortality process error SD	Normal(0,5)[0,∞]
$M_{\text{init}}$	Initial (year‐1) log natural mortality rate	Normal(0,5)
${F_{B}}_{\text{init}}$	Initial (year‐1) log bycatch mortality rate	Normal(0,7.5)
$\lambda_{\text{init}}$	Initial (year‐1) log maturation schedule	Normal(1,1) [0,∞]
${F_{T}}_{\text{init}}$	Initial (year‐1) log harvest mortality rate	Normal(0,5)
${\mu_{G}}_{a}$	Among‐year mean of logit bycatch stock composition‐at‐age	Normal(0,5)
$\;{\sigma_{G}}_{a}$	Among‐year SD of logit bycatch stock composition‐at‐age	Normal(0,5) [0,∞]

*Note*: Values in parentheses indicate the mean and SD of the specified prior distribution, and brackets indicate upper and lower bounds. Priors without brackets indicate that no bound was placed on these parameters.

### Retrospective simulations

To illustrate the potential roles of harvest/bycatch removals and variation in key life history processes (i.e., juvenile recruitment, natural mortality) in driving realized declines of Canada‐origin Yukon River Chinook salmon abundance over our study timeframe, we used model fits of the IPM to conduct a series of retrospective simulations. In these simulations, juvenile recruitment errors, post‐juvenile natural mortality, bycatch, and harvest were sequentially manipulated, while maintaining all other parameters at their estimated posterior values. For a given factor (i.e., bycatch, harvest, recruitment, or natural mortality), the corresponding retrospective simulation is intended to represent an alternative scenario in which potential negative impacts of that factor on past abundance trends are reduced or eliminated altogether. By comparing the resulting adult abundance (i.e., run size; $A_{y}=\sum_{a=4}^{a=7}A_{y,a}$) trajectories from these retrospective simulations to that of the fitted model, we illustrate how estimated variation in juvenile recruitment and natural mortality, as well as bycatch and harvest removals, have contributed to realized declines in run sizes over our study period.

#### Bycatch removals

An important consideration for managing Chinook salmon bycatch in the EBS pollock fishery has been to determine its impacts on the realized abundance of adult fish returning to their natal river (Ianelli & Stram, 2015). Not all bycaught fish would necessarily have survived to maturity had they escaped capture by the pollock fleet such that the estimates of natural mortality $\left(M_{y}\right)$ and maturation $\left(\theta_{y_{c},a}\right)$ rates generated by the IPM can be leveraged towards understanding the impacts of bycatch removals on adult abundance. Similarly, it is also important to consider how such reductions in run size might propagate to influence future returns via impacts on juvenile production, which will vary with spawner abundance due to density dependence as governed by the spawner‐recruit function, and over time due to temporal variability in recruitment errors. As such, to understand the long‐term effects of bycatch removals, we simulated an alternative population trajectory from the posterior of the fitted model in which the bycatch mortality rate ($F_{B}$) was fixed at zero in all years. For each simulation replicate, all other parameters were set equal to their estimated values in the corresponding posterior sample from the fitted model. Trajectories of total adult abundance over time were compared between the fitted model (including bycatch removals) and the simulation output (without bycatch removals) to illustrate the extent to which bycatch has contributed to realized variation in run size over time.

#### Harvest

In addition to estimating bycatch impacts, a similar retrospective simulation approach can be used to understand the effects of modeled harvest on adult abundance trends. To characterize harvest impacts, we simulated an alternative population trajectory in which the terminal harvest mortality rate ($F_{T}$) was fixed at zero, and compared the resulting run size trajectory from these simulations (no harvest) to that from the fitted model (with harvest).

#### Juvenile recruitment and post‐juvenile natural mortality

While it is relatively straightforward to characterize the impacts of bycatch and harvest removals in a retrospective simulation context by effectively “turning off” these sources of mortality (i.e., setting $F_{B}$ or $F_{T}$ to zero), alternative juvenile recruitment and post‐juvenile natural mortality scenarios to consider are less readily apparent. However, as described below (see *Post‐juvenile natural mortality*), natural mortality exhibited limited temporal variation from 2003 to 2015, but began increasing substantially after 2016. To explore the impacts of such elevated natural mortality, we simulated an alternative population trajectory in which the 2016–2023 natural mortality rates were replaced with the median pre‐2016 mortality level, effectively representing a scenario where this rise in natural mortality did not occur. We compared adult abundance trends from these simulations to those of the fitted model to illustrate the impacts of elevated natural mortality on recent run sizes.

While the clear shift in natural mortality after 2016 points to a plausible baseline mortality scenario to compare to in our retrospective simulations, there is no such readily apparent baseline for juvenile recruitment. As such, we generated an alternative “favorable juvenile recruitment” scenario by fixing recruitment deviations (${\varepsilon_{R}}_{y}$) in all years at the 95th percentile value of median ${\varepsilon_{R}}_{y}$ estimates. Recruitment deviations of this approximate magnitude occurred in early years (e.g., 2003, 2005) of our study period and during a series of strong recruitment events from 2013 to 2016 that preceded a partial recovery in adult abundance from 2016 to 2019 (see *Juvenile recruitment*), and thus may more closely resemble historical (i.e., pre‐decline) juvenile recruitment strength. We compared adult abundance trends from these retrospective simulations to those of the fitted model to illustrate the degree to which consistently favorable juvenile recruitment would (or would not) have ameliorated the realized declines in run sizes that occurred across our study period. Finally, we also conducted retrospective simulations specified with favorable juvenile recruitment (i.e., ${\varepsilon_{R}}_{y}$ fixed at their 95th percentile value) and no increase in natural mortality (i.e., natural mortality rates from 2016 to 2023 fixed at the median pre‐2016 level).

### Forward projections

In addition to retrospective simulations, the IPM can also be used to project population dynamics into the future. In such forward projections, future values of the post‐juvenile natural mortality ($M_{y}$) and maturation ($\lambda_{y_{c}}$) terms were simulated following random‐walk processes as specified in the fitted model, with new process errors (${\varepsilon_{M}}_{y},{\varepsilon_{\lambda }}_{y}$) generated from their corresponding estimated distributions (i.e., $\text{Normal}\left(0,\sigma_{\lambda }\right)$, $\text{Normal}\left(0,\sigma_{M}\right)$). While bycatch mortality ($F_{B}$) was also specified as a random walk in the fitted model, Chinook salmon bycatch in the EBS pollock fishery has been constrained by a system of hard caps and industry‐led incentive plan agreements introduced under Amendment 91 to the Bering Sea and Aleutian Islands (BSAI) Groundfish Fishery Management Plan in 2011 (NMFS, 2010). Consequently, bycatch levels occurring prior to 2011 are unlikely to occur again under current regulations such that the bycatch process error distribution from the fitted model may not be representative of plausible future values. Moreover, Chinook salmon bycatch in the EBS pollock fishery has remained at relatively stable levels since 2011 (DeFilippo et al., 2025). Therefore, bycatch mortality was fixed at its mean estimated post‐Amendment 91 (2011–2023) value in all projections. The terminal harvest mortality rate ($F_{T}$) was fixed at zero in all forward projections. Future recruitment deviations (${\varepsilon_{R}}_{y}$) were generated from their estimated distribution in the fitted model ($\text{Normal}\left(0,\sigma_{R}\right))$. Forward projections were generated over 7 years (2024–2030), encompassing the maximum time for Yukon River Chinook salmon to complete one life cycle. To understand the biological conditions conducive to recovery, we compared the simulated values of relevant parameters, including Ricker spawner‐to‐juvenile productivity (α) and capacity ($\beta^{-1}$), recruitment deviations (${\varepsilon_{R}}_{y}$), and natural mortality ($M_{y}$), between projection iterations in which final (2030) abundance levels were greater than or equal to the 90th percentile of the projected 2030 run size distribution ($A_{y=2030}\geq$48,500) versus those that resulted in abundance remaining near or below recent low levels ($A_{y=2030}\leq$16,000). To explore the sensitivity of our results to the length of the projection window, we also explored projections over a 14‐year (two maximum life cycles) time horizon (Appendix S1: Figure S1). While projections may to some extent be sensitive to structural assumptions of the IPM (e.g., the random‐walk specification of natural mortality vs. temporally independent recruitment errors), we also simulated projections with fixed recruitment errors (${\varepsilon_{R}}_{y}$) and natural mortality rates ($M_{y}$) to explicitly consider how these processes may interact to shape recovery outcomes (Appendix S1: Figure S2).

## RESULTS

### Juvenile recruitment

**FIGURE 3:**
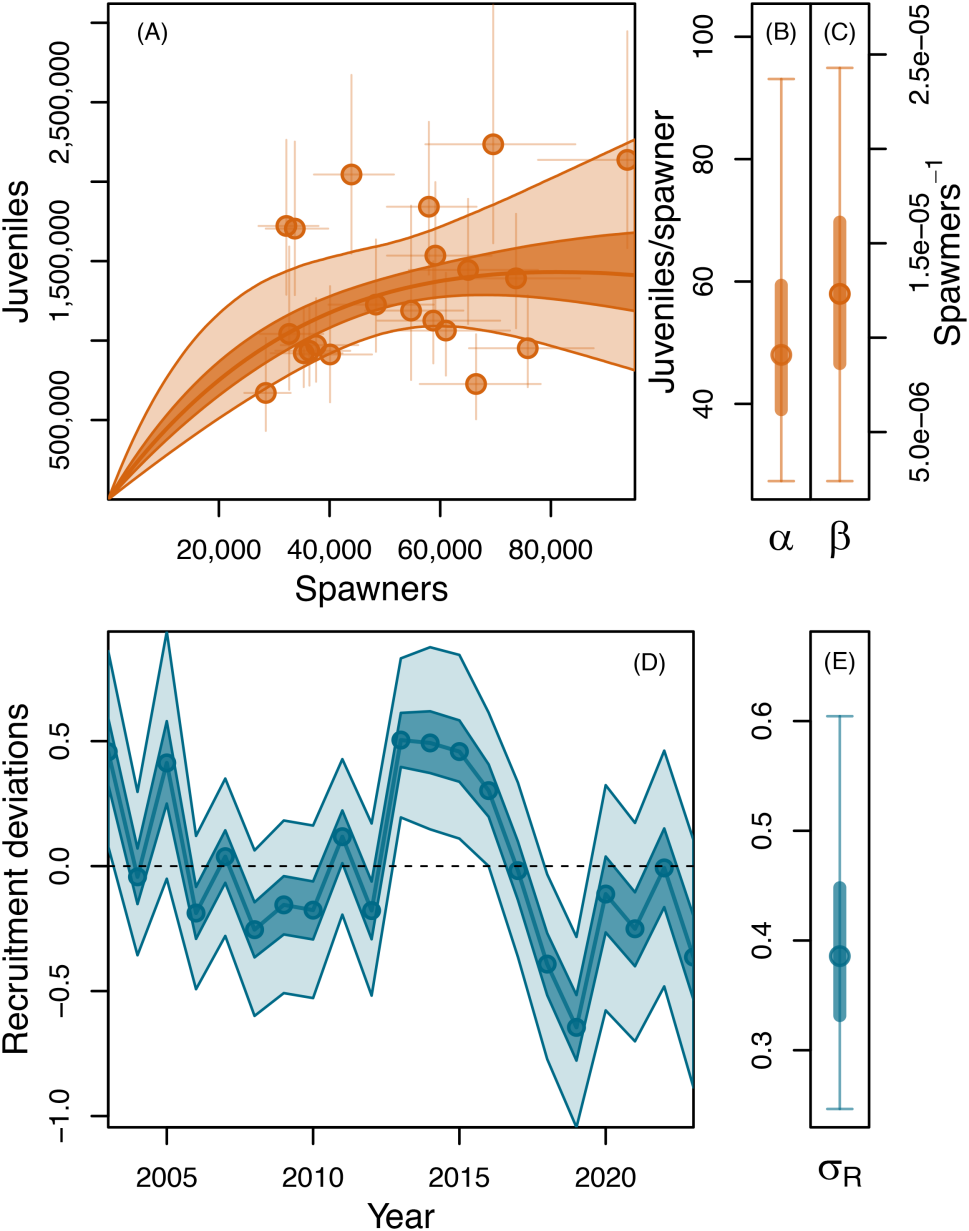
Canada‐origin Yukon River Chinook salmon spawner‐to‐juvenile recruitment patterns. Panel (A) shows the estimated Ricker spawner‐recruit curve, with circles and associated lines indicating medians and 95% credible intervals of spawner/juvenile abundance. Panels (B) and (C) show the estimated Ricker productivity (α) and (inverse) capacity (β) parameters. Panel (D) shows the time series of deviations from the estimated stock‐recruit relationship ($\varepsilon_{R}$), and panel (E) shows the estimated posterior distribution of the SD of the recruitment error distribution ($\sigma_{R}$). In panels (A) and (D), Solid lines represent median values, and 50% and 95% credible intervals are indicated by dark‐ and light‐shaded boundaries, respectively. In panels (B, C, and E) posterior median values are indicated by filled circles, and 50% and 95% credible intervals are indicated by thick and thin lines, respectively.

**FIGURE 4:**
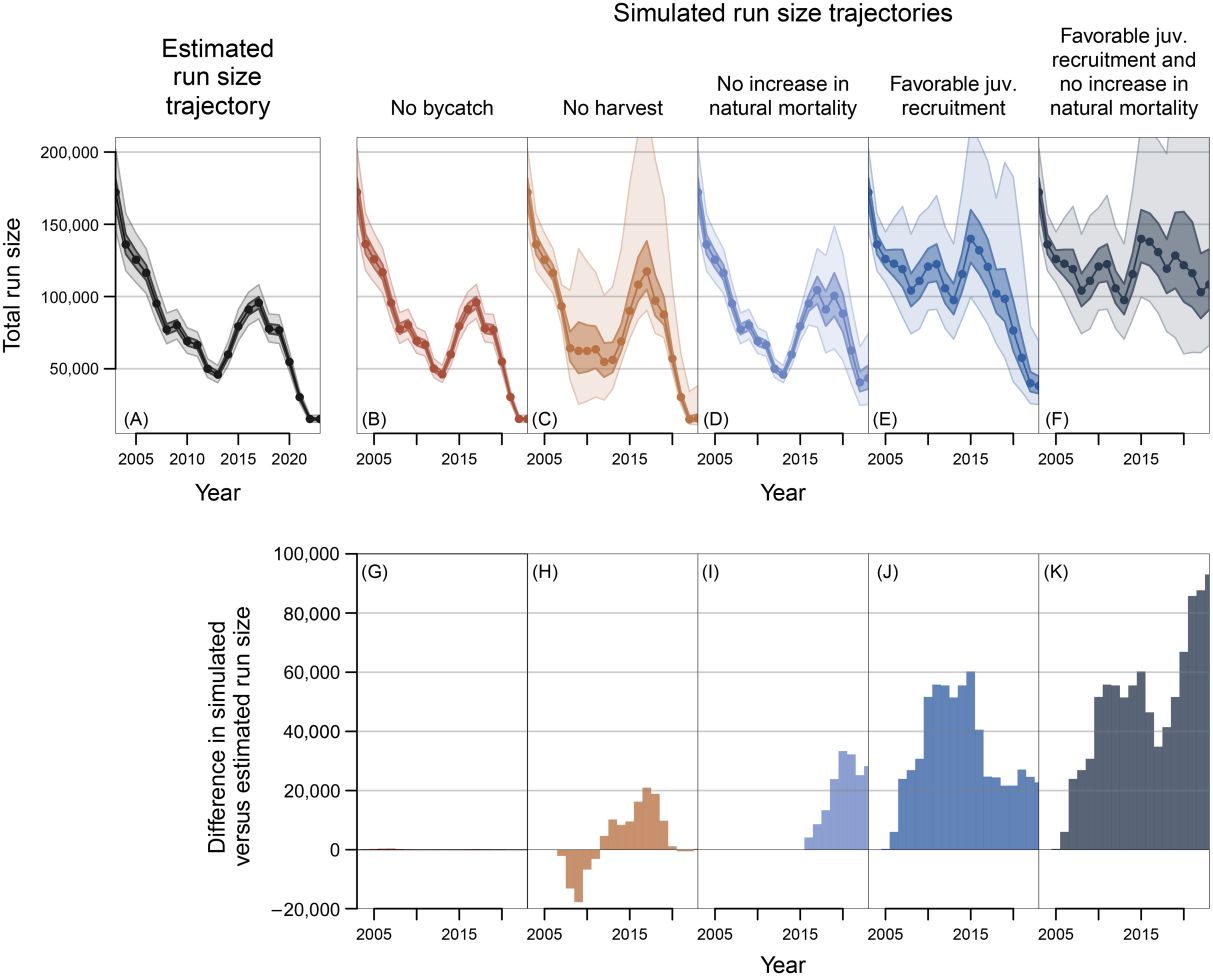
Retrospective simulation outcomes. Panel (A) shows the posterior estimates of upper (Canada‐origin) Yukon River Chinook salmon run sizes from 2003 to 2023 from the fitted model. Panel (B) shows the run size trajectory from retrospective simulations without bycatch (${F_{B}}_{y}=0$). Panel (C) shows the run size trajectory in simulations without harvest (${F_{T}}_{y}=0$). Panel (D) shows the run size trajectory in simulations where post‐juvenile natural mortality rates ($M_{y}$) from 2016 to 2023 were fixed at the median pre‐2016 level. Panel (E) depicts the run size trajectory for simulations with consistently favorable juvenile recruitment (${\varepsilon_{R}}_{y}$ fixed at their 95th percentile). Panel (F) shows the run size trajectory from simulations specified with both favorable recruitment and no elevated 2016–2023 natural mortality rates. Bars in panels (G–K) show the median differences in run size between the fitted model (A) and the simulations depicted in panels (B–F), respectively. In panels (A–F), median values are represented by solid lines and circles, and 50% and 95% credible intervals are represented by dark‐ and light‐shaded boundaries, respectively.

Estimated posterior distributions for the Ricker spawner‐recruit parameters indicated a median value for maximum spawner‐to‐juvenile productivity at low spawning stock size (α) of 48 juvenile per spawner (95% credible interval [CI] = 27–93 juveniles per spawner) and a median capacity ($\beta^{-1}$) of 81,178 spawners (95% CI = 41,172–416,270 spawners) (Figure 3A–C). Juvenile recruitment deviations (${\varepsilon_{R}}_{y})$ varied considerably over time, exhibiting a period of mostly negative values from 2006 through 2012 (with exceptions in 2007 and 2011), followed by a string of strong recruitment events from 2013 to 2016 (Figure 3D), which likely contributed to the partial recovery in adult run sizes from 2016 to 2019 (Figures 1B and 4A) when many of the juveniles from these year‐classes reached maturity. More recent recruitment errors from 2017 to 2023 were negative or neutral (near‐zero) (Figure 3D).

Retrospective simulations characterized by consistently favorable juvenile recruitment deviations (i.e., ${\varepsilon_{R}}_{y}$ fixed at their 95th percentile value) did not exhibit similar declines in run size as observed in the fitted model during the early portion of our study period (cf. Figure 4A with Figure 4E,J). While abundance still dropped sharply in the initial years of the retrospective simulations (Figure 4E), these runs were partially or entirely produced by recruitment events that occurred prior to our study's timeframe and thus were less influenced, or unaffected altogether, by the favorable recruitment errors specified in our simulations. Crucially, even in retrospective simulations with favorable juvenile recruitment in all years, run sizes still declined substantially towards the end of our study period (Figure 4E).

### Post‐juvenile natural mortality

**FIGURE 5:**
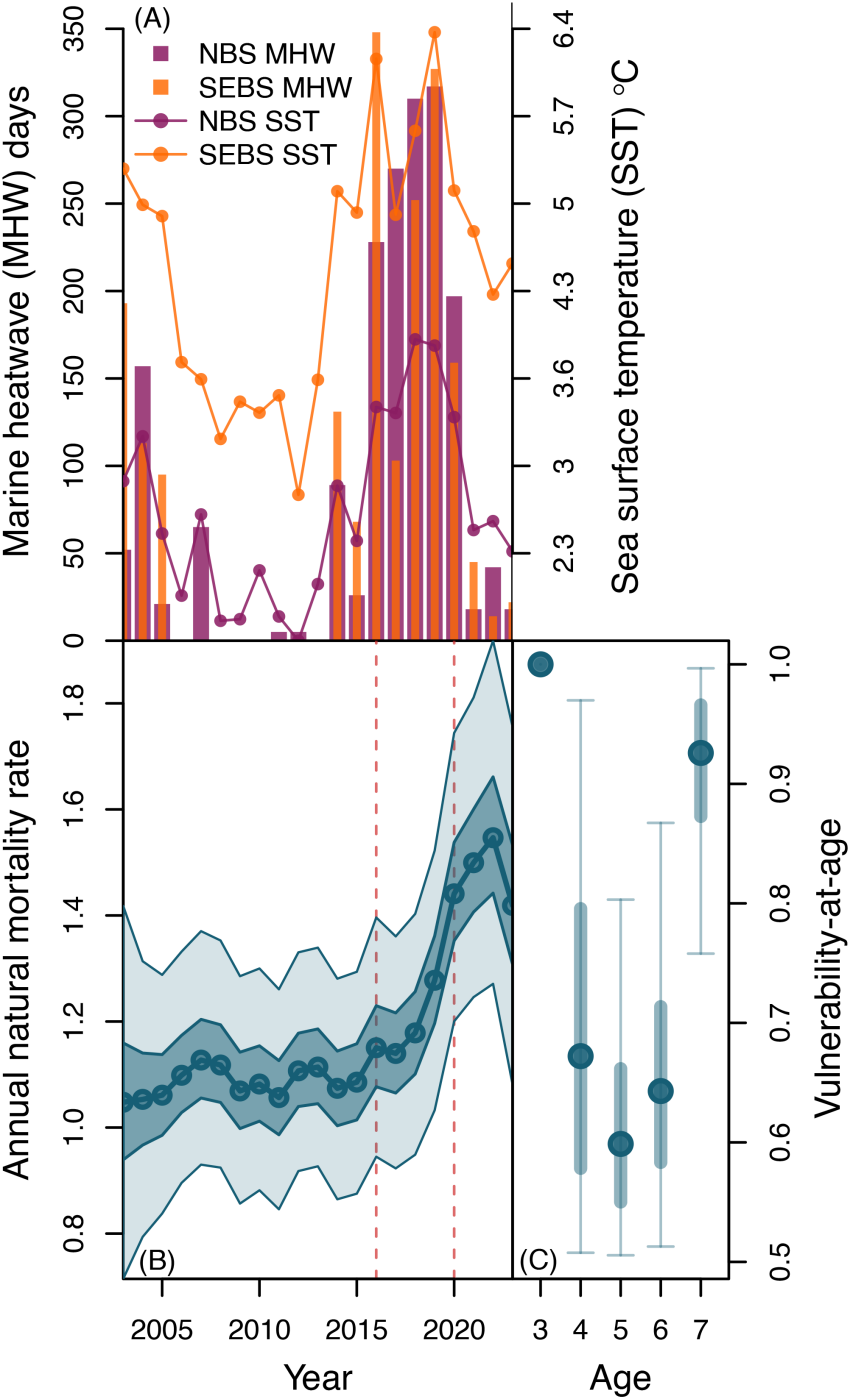
Post‐juvenile natural mortality patterns. Panel (A) shows the number of days characterized by marine heatwave conditions based on the Hobday et al. (2016) classification for the Northern Bering Sea (NBS) (purple, thick bars) and the southeastern Bering Sea (SEBS) (orange, thin bars), and the annual average satellite‐derived sea surface temperature for the NBS (purple dots/lines), and SEBS (orange dots/lines). Note that bars for the SEBS and NBS marine heatwave days are overlaid, not stacked. Panel (B) shows posterior estimates of annual post‐juvenile natural mortality $\left(M_{y}\right)$ over time, with median values indicated by circles and lines, and 50% and 95% credible intervals shown as dark‐ and light‐shaded boundaries, respectively. The 2016–2020 marine heatwave period is marked in panel (B) by dashed vertical red lines. Panel (C) shows the estimated posterior distributions of vulnerability‐at‐age to natural mortality ($v_{a}$), with medians indicated by filled circles, and 50% and 95% credible intervals indicated by thick and thin lines, respectively.

Post‐juvenile natural mortality rates $(M_{y}$) exhibited limited temporal variation during the early portion of our study period, with median estimates ranging between 1.05 and 1.13 (equivalent to 32%–35% survival) from 2003 to 2015 (Figure 5B). However, in subsequent years (2016–2023), natural mortality increased, reaching peak levels in 2020–2023 with median estimates of 1.42–1.55 (equivalent to 21%–24% survival) (Figure 5B). This rise in natural mortality occurred following the onset of an acute marine heatwave period in the Bering Sea spanning from ~2016 to 2020 (Figure 5A). Estimates of vulnerability‐at‐age to natural mortality were highly uncertain, but generally declined from age‐3 (fixed at 1) through ages 4 (median = 0.67, 95% CI = 0.51–0.97) and 5 (median = 0.60, 95% CI = 0.51–0.80) before increasing slightly for age‐6 (median = 0.64, 95% CI = 0.51–0.87) and more substantially for age‐7 fish (median = 0.93, 95% CI = 0.76–1.0) (Figure 5C).

Retrospective simulations in which the post‐2016 rise in natural mortality did not occur exhibited large differences in run size from the fitted model for the latter portion of our study period (cf. Figure 4A with Figure 4D). In particular, from 2019 to 2023, median run sizes in retrospective simulations without the elevated post‐2016 mortality rates were substantially higher (by >23,000 to >33,000 fish) compared to the fitted model (Figure 4I). However, while these retrospective simulations resulted in higher run sizes by the end of our study period, they still exhibited substantial declines in abundance (Figure 4D). Conversely, retrospective simulations with both favorable juvenile recruitment and no increase in natural mortality resulted in comparatively stable abundance levels that did not exhibit the long‐term declines observed in the fitted model (cf. Figure 4A with Figure 4F,K). These retrospective simulations indicate that the most recent and particularly acute drop in adult abundance likely resulted from years of both low juvenile recruitment and increased post‐juvenile natural mortality.

### Bycatch and harvest

**FIGURE 6:**
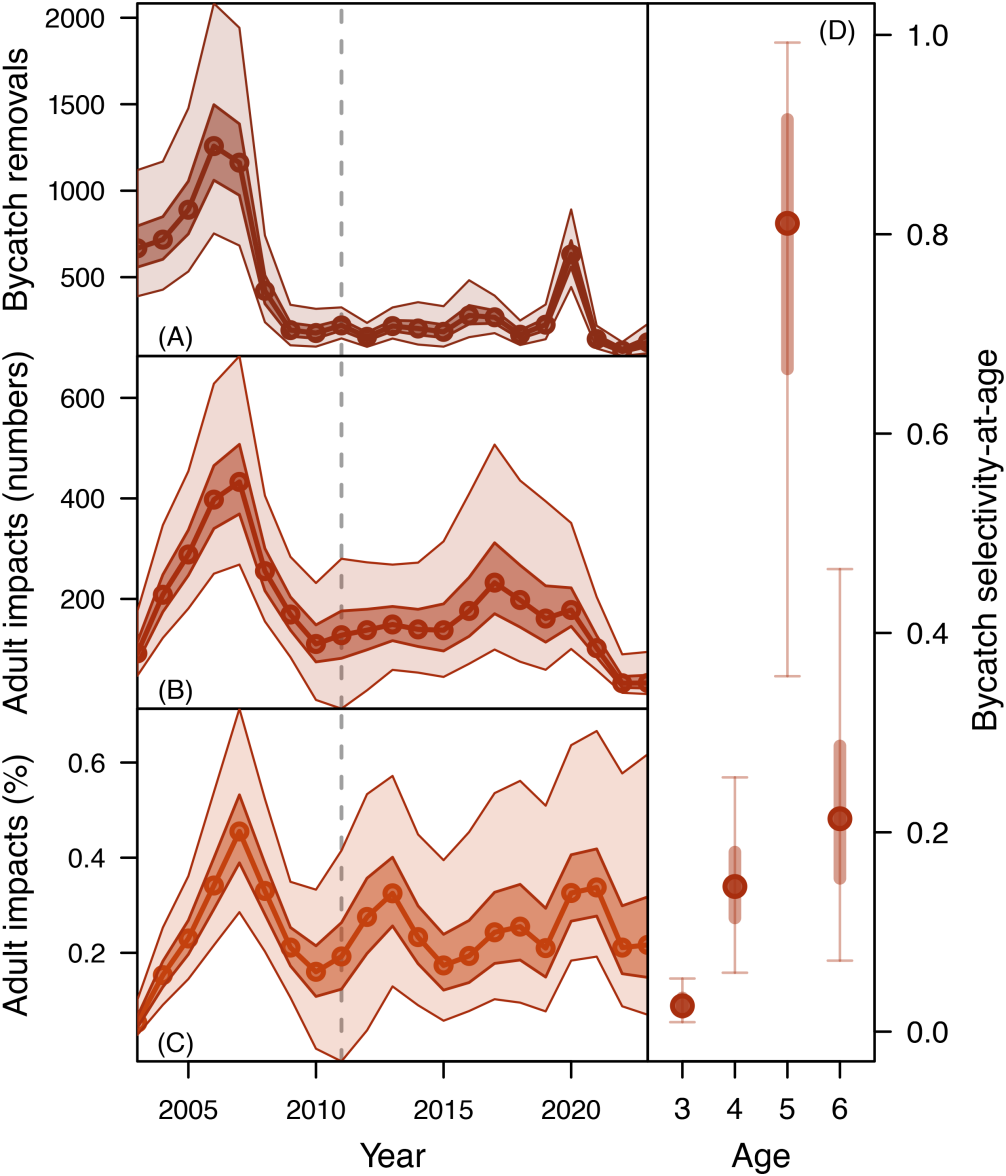
Patterns of upper (Canada‐origin) Yukon River Chinook salmon bycatch in the eastern Bering Sea (EBS) pollock fishery. Panel (A) shows the estimated abundance of Canada‐origin Yukon River Chinook salmon bycatch removals by year, while panels (B) and (C) show the estimated impact of these removals (based on retrospective simulations where ${F_{B}}_{y}=0$) on adult run size by return year in terms of abundance (B) and percent of run size (C). The implementation of Amendment 91 in 2011, which imposed Prohibited Species Catch (PSC) limits on Chinook salmon bycatch in the EBS pollock fishery, is indicated by a dashed gray line in panels (A–C). In panels (A–C), median estimates are indicated by circles and lines, while dark‐ and light‐shaded boundaries represent the 50% and 95% credible intervals. Panel (D) shows the estimated bycatch selectivity‐at‐age, with median values shown as filled circles and 50% and 95% credible intervals shown as thick and thin lines, respectively. Note that no age‐2 or age‐7 Chinook salmon encountered by the EBS pollock fishery were genetically assigned to the Canada‐origin Yukon River reporting group during our study period, and so selectivity was not estimated for these age classes.

Estimated bycatch of Canada‐origin Yukon River Chinook salmon across our study period was greatest in 2006, with a median estimated removal of 1258 fish (95% CI = 753–2083), and reached its lowest level in 2022 (median estimated catch = 76 fish 95% CI = 44–125) (Figure 6A). Since the implementation of Amendment 91 in 2011, annual median estimates of Canada‐origin bycatch removals have averaged 223 fish (Figure 6A). Bycatch selectivity increased with age from a median estimate of 0.03 (95% CI = 0.01–0.05) for age‐3 fish to 0.15 (95% CI = 0.06–0.26) and 0.81 (95% CI = 0.36–0.99) for age‐4 and age‐5 fish, respectively, but declined to 0.21 (95% CI = 0.07–0.46) for age‐6 fish (Figure 6D). While bycatch removals have been relatively stable in recent years (particularly since the implementation of Amendment 91 in 2011), in‐river harvest has been more variable. Loosely tracking variation in run size, harvest declined from 2003 to 2014, increased moderately from 2015 to 2020, and dropped substantially from 2021 to 2023 (Appendix S2: Figure S1).

Retrospective simulations with zero bycatch removals ($F_{B}=0$) resulted in marginally higher run sizes compared to the fitted model that included bycatch removals (Figure 4B,G). The greatest difference occurred in 2007, in which the median run size from the zero‐bycatch simulations was 433 fish greater than that of the fitted model (Figure 6B). In other years, median differences in run size between the fitted model and zero‐bycatch simulations ranged from 32 to 398 fish (Figure 6B). However, these differences did not qualitatively alter the simulated run size trajectory (cf. Figure 4A with Figure 4B). Retrospective simulations without directed harvest ($F_{T}=0$) resulted in mixed differences in run size over time compared to the fitted model. In the early years of our study period, when abundance levels were higher, lack of directed fishing negatively impacted run sizes compared to the fitted model (Figure 4C,H), likely due to overcompensation implied by the Ricker spawner‐recruit function at greater escapement levels. Despite limited overcompensation within the range of observed escapements (Figure 3A), run sizes and associated catches were relatively large during the early portion of our study period such that lack of directed fishing during this time resulted in larger simulated escapement levels at which overcompensation was more pronounced (Appendix S1: Figure S3). In subsequent years, when run sizes were typically lower, the zero‐harvest simulations exhibited modestly greater abundance compared to the fitted model, with positive differences ranging from ~4700 to ~21,000 fish from 2012 to 2019 (Figure 4C,H). However, the general run size trajectory of the zero‐harvest simulations was similar to that of the fitted model, and the simulated population still declined to comparably low levels as seen in the fitted model by the end of our study period (Figure 4C,H).

### Projection outcomes

**FIGURE 7:**
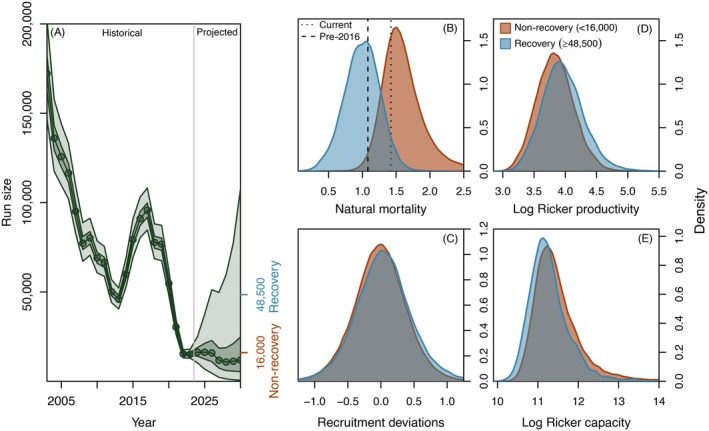
Forward projection outcomes. Panel (A) shows the historical and projected future trajectory of Canada‐origin Yukon River Chinook salmon run sizes. The gray vertical line demarcates the beginning of the projection window (2024–2030). Median values are shown as circles and solid lines, while 50% and 95% credible intervals are indicated by dark‐ and light‐shaded boundaries, respectively. Panels (B–E) show the distributions of parameter values in projection iterations that resulted in final (2030) run sizes greater than or equal to the 90th percentile (≥48,500 fish) of the projected final run size distribution (blue), versus those that resulted in run sizes remaining near or below recent low abundance levels (<16,000 fish, brown). Note that Ricker capacity (panel E) is plotted in log space to aid visualization. In panel (B), the dashed vertical line indicates the median pre‐2016 (pre‐heatwave) natural mortality rate, while the most recent 2023 (i.e., “current”) median estimate of natural mortality is shown by the dotted line. Simulations shown here were specified with zero harvest, and bycatch mortality fixed at the mean estimated post‐Amendment 91 level.

Seven‐year forward projections exhibited substantial variability in final (2030) abundance levels (Figure 7A). We compared the distributions of key parameters between projections resulting in final run sizes that were greater than or equal to the 90th percentile (≥48,500) of the projected 2030 run size distribution ($A_{y=2030}$) versus those in which final run sizes remained near or below recent low levels (i.e., ≤16,000). For brevity, we refer to these projection outcomes and associated parameter spaces as “recovery” $\left(A_{y=2030}\geq 48,500\right)$ and “non‐recovery” ($A_{y=2030}$ ≤16,000) scenarios. There were limited differences between the distributions of simulated juvenile recruitment deviations (${\varepsilon_{R}}_{y}$) between recovery and non‐recovery projection scenarios (Figure 7C). The distributions of the Ricker spawner‐recruit terms in the recovery projections were slightly skewed towards greater spawner‐to‐juvenile productivity (α) and lower capacity ($\beta^{-1}$) compared to the non‐recovery projections (Figure 7D,E), likely reflecting negative correlations between these terms in the joint posterior of the fitted model. The clearest difference in parameter values between the recovery and non‐recovery projections was a tendency for lower post‐juvenile natural mortality draws ($M_{y}$) in the former (Figure 7B). Indeed, 87% of projections in which simulated natural mortality rates were consistently at or below the median pre‐2016 mortality level resulted in 2030 run sizes greater than or equal to the 90th percentile of the final run size distribution (i.e., $A_{y=2030}\geq 48,500$). Conversely, of the projections in which natural mortality consistently remained at or above the most recent (2023) median estimate, <0.1% resulted in 2030 run sizes greater than or equal to the 90th percentile of the final run size distribution. In projections carried out over longer time horizons (i.e., 14 years), post‐juvenile natural mortality exhibited similar importance in shaping recovery outcomes (Appendix S1: Figure S1).

## DISCUSSION

In this study, we developed an IPM to explore stage‐specific constraints on population productivity and recovery potential for Canada‐origin Yukon River Chinook salmon. Our results point to periods of low juvenile recruitment and a recent increase in post‐juvenile natural mortality as important contributors to declining abundance levels across our study period. Conversely, our findings indicate comparatively limited impacts of harvest and bycatch removals. The role of juvenile recruitment we describe is consistent with previous studies that have implicated factors such as reduced fecundity and adverse environmental impacts on freshwater/early marine survival as likely contributors to dwindling run sizes (Cunningham et al., 2018; Feddern et al., 2024; Murdoch et al., 2023; Ohlberger et al., 2020). However, our study also indicates that the most recent and particularly acute drop in adult abundance from 2019 to 2023 is not attributable to poor juvenile recruitment alone, but reflects the impacts of elevated post‐juvenile natural mortality as well, representing an apparent shift in the critical life history stages and processes therein acting to constrain productivity.

Post‐juvenile natural mortality of Yukon River Chinook salmon has previously been considered to be largely stable, as evidenced by the predictive skill of the NBEST survey's juvenile abundance estimates for forecasting future adult returns (Murphy et al., 2017). Similarly, our results indicated limited variation in natural mortality during the early portion of our study period (2003–2015). However, estimates of post‐juvenile natural mortality rates rose considerably from ~2016 to 2020 following the onset of a pronounced marine heatwave period that precipitated wide‐ranging shifts in Bering Sea ecosystem conditions (Carvalho et al., 2021; Siddon, 2022; Siddon & Zador, 2019). Our results indicate that such elevated natural mortality rates, combined with several years of weak juvenile recruitment, led to the particularly acute downturn in run sizes from 2019 to 2023. While natural mortality of salmon is typically expected to decline with increasing size and age (Beamish & Mahnken, 2001; Riddell et al., 2018), the IPM's estimates of vulnerability‐at‐age to natural mortality rose slightly from age‐5 to age‐6, and more substantially from age‐6 to age‐7. Such increased vulnerability estimates for older fish could potentially reflect size‐selective consumption by marine predators such as salmon sharks (*Lamna ditropis*) and killer whales (*Orcinus orca*), which can represent sources of late‐stage ocean mortality for Chinook salmon (Manishin et al., 2021; Ohlberger et al., 2019; Seitz et al., 2019). Alternatively, the heightened vulnerability to natural mortality estimated for older fish may simply be a model artifact arising from lower returns of these individuals than expected under the logistic maturation schedule, which the IPM may have compensated for with elevated late‐stage mortality. Future research exploring more flexible forms of the maturation and mortality sub‐models may help resolve any such discrepancies, although we note that the relative abundance of age‐7 fish in the population is low (Appendix S1: Figure S4) such that mortality estimates for these individuals may be uncertain regardless of model specification, but likely had limited qualitative impact on our findings.

The Bering Sea exhibited widespread shifts in ecosystem structure and function during and following the recent marine heatwave period that could have contributed to increased post‐juvenile mortality of Chinook salmon (Overland et al., 2024; Siddon & Zador, 2019). For instance, declines in the biomass of forage fishes such as capelin and herring, and the energetic content of juvenile pollock—each of which are important prey items for Chinook salmon—were documented in association with recent marine heatwave conditions in the Bering Sea (Andrews et al., 2024; Page et al., 2024; Suryan et al., 2025; Yasumiishi et al., 2020). Importantly, such reductions in forage fish biomass and juvenile pollock energy density persisted even after heatwave conditions abated and temperatures began returning to near‐average levels, providing a possible explanation for why our estimates of natural mortality remained elevated in years following the end of the heatwave period (Andrews et al., 2024; Page et al., 2024; Suryan et al., 2025). Persistent impacts from marine heatwaves have been similarly described in adjacent ecosystems (i.e., the Gulf of Alaska; Suryan et al., 2021), and heatwave‐driven shifts in marine food webs have been suggested to adversely affect Chinook salmon in other portions of their distribution (Crozier et al., 2025; Gomes et al., 2024). Moreover, other forage fish predators were also negatively impacted during/following the heatwave years, with indications of a potential concurrent rise in natural mortality of Pacific cod (*Gadus macrocephalus*) (Spies et al., 2024), as well as reduced body condition of seals (Boveng et al., 2020), and mortality and reproductive failure of seabirds (Jones et al., 2019; Renner et al., 2024). Similarly, heightened predation pressure and/or competition driven by heatwave conditions and associated food web disruptions could also have contributed to increased mortality of Chinook salmon (Crozier et al., 2025).

While the elevated post‐juvenile natural mortality we detected in recent years may represent losses during marine residency, it could also reflect in‐river adult mortality (see *Model inputs*). Factors such as disease (e.g., infections by the parasite *Ichthyophonus*) and thermal and metabolic stress experienced during freshwater spawning migrations have received increasing attention as potentially important sources of mortality (Hamazaki et al., 2013; Kocan et al., 2004; Von Biela et al., 2020, 2022), and may have been particularly acute in recent years (Howard & von Biela, 2023; Jallen et al., 2022). Lower prey availability/quality in the marine environment during and following the recent heatwave period could also have contributed to in‐river adult mortality via reduced post‐ocean body condition and energetic reserves for undertaking lengthy spawning migrations, possibly interacting with factors such as disease and direct thermal/metabolic stress experienced in freshwater (Howard & von Biela, 2023). Similarly, heatwave‐driven shifts in marine food webs could have influenced the prevalence of *Ichthyophonus* infections in Yukon River Chinook salmon, which are thought to contract the pathogen from prey consumed during ocean residency (Murphy et al., 2023; White et al., 2014). Future developments in explicitly estimating and accounting for in‐river adult mortality would likely be informative for further understanding its role in Yukon River Chinook salmon population dynamics and for distinguishing post‐juvenile mortality occurring in the marine environment from losses incurred during freshwater spawning migrations.

Regardless of its causes, our results indicate that elevated post‐juvenile natural mortality has emerged as an important constraint on contemporary productivity dynamics, acting in conjunction with low juvenile recruitment to drive the most recent and precipitous drop in abundance. The significance of post‐juvenile natural mortality is particularly evident from our projection analyses, in which it exerted a strong limiting influence on recovery outcomes. Indeed, we found that substantive recovery was unlikely in scenarios where recent, elevated levels of natural mortality persisted into the future, while recovery potential was improved in projections where natural mortality rates returned to or below their median pre‐2016 level. Although compensatory increases in per‐capita juvenile recruitment at low spawner abundance would typically be expected to promote recovery, our results suggest that the emergence of a novel mortality bottleneck in the post‐juvenile life stages can countervail such effects, thereby limiting recovery potential (e.g., Kuparinen & Hutchings, 2014). We can explore the interaction between natural mortality and juvenile recruitment strength explicitly by fixing them at pre‐specified values in our model projections (Appendix S1: Figure S2); even with consistently favorable juvenile recruitment (i.e., ${\varepsilon_{R}}_{y}$ fixed at their 95th percentile value in all years from 2024 to 2030), increases in run sizes were minor if recent (i.e., 2023), elevated natural mortality levels persisted throughout the projection window (Appendix S1: Figure S2). Conversely, recovery outcomes improved if natural mortality during the projection period was fixed at its median pre‐2016 rate, even at average levels of juvenile recruitment (i.e., ${\varepsilon_{R}}_{y}$ fixed at 0 from 2024 to 2030) (Appendix S1: Figure S2). Thus, it appears that the elevated post‐juvenile natural mortality levels in recent years are of sufficient magnitude to represent a bottleneck to population productivity, effectively limiting recovery potential even in the presence of improved juvenile output.

There are several caveats and limitations to our study that bear consideration. Given that our model fits did not include data from the 1980s and early to mid‐1990s, when Yukon River Chinook salmon were at higher abundance levels, our parameter estimates may not encompass the full scope of historical variability. For instance, if long‐term declines in spawner‐to‐juvenile recruitment strength have occurred (e.g., due to reduced freshwater/early marine survival and/or fecundity; Feddern et al., 2024; Cunningham et al., 2018; Ohlberger et al., 2020), then contemporary periods of per‐capita spawner‐to‐juvenile output resembling typical historical levels would likely manifest as positive recruitment anomalies. This could explain why only retrospective simulations with strongly positive juvenile recruitment errors in all years (in conjunction with pre‐2016 natural mortality) maintained run sizes at relatively stable levels. Likewise, the results of our forward projection analyses were constrained by the range of parameter values (and associated uncertainty) estimated for our study period, and thus did not account for potential variability beyond these levels. Moreover, while our IPM accounted for declines in age‐at‐maturity via a time‐varying maturation schedule, we did not explicitly link such demographic shifts to population productivity (e.g., via reductions in body size and impacts on fecundity), which we recommend as a direction for future model developments. Finally, while our results indicated a limited role of bycatch removals in driving realized declines in run size, it should be noted that the Canada‐origin Yukon River stock comprises a minor share of the EBS pollock fishery's Chinook salmon bycatch compared to some other genetic reporting groups (e.g., Coastal Western Alaska; Guthrie et al., 2022). Thus, our findings may not necessarily be representative of bycatch impacts for other stocks. Additionally, our zero‐bycatch retrospective simulations assumed that additional fish surviving to maturity in the absence of bycatch would still be subject to directed harvest as adults (based on the estimated harvest mortality rate in the year that they matured). As such, these simulations may underestimate impacts on future juvenile production compared to a scenario where all of fish that survived to maturity in the absence of bycatch escaped harvest and were allowed to spawn.

This study offers practical insights that can help inform conservation and fisheries management efforts. Our findings suggest that extreme environmental events, such as marine heatwaves, have the potential to induce novel stage‐specific survival bottlenecks, reshaping the key life history periods and processes therein acting to constrain population productivity. Ecosystem disruptions from marine heatwaves are widespread (Smale et al., 2019; Smith et al., 2023; Suryan et al., 2021) such that the patterns we describe in this study may be broadly applicable to Chinook salmon throughout their distribution (Crozier et al., 2025; Gomes et al., 2024), and possibly to other species as well (Farley et al., 2024). Given that marine heatwaves are expected to become more frequent and severe with continued warming (Frölicher et al., 2018), similar rises in mortality—and concomitant limitation of productivity and recovery potential—as described here could become increasingly common in the future. Therefore, it may be advantageous to explore developing management procedures that can adaptively respond to such episodic perturbations (Bessell‐Browne et al., 2025; Caputi et al., 2016), which we recommend as a focus for future work. As salmon face rapidly changing conditions, there is a growing need to understand how environmental and anthropogenic impacts across the life cycle interact to drive variation in productivity (Alaska Salmon Research Task Force, 2024). Consequently, integrated stage‐structured models represent valuable research tools for informing management of salmon stocks that is robust to ecosystem change and supports the well‐being of communities that depend on them.

## CONFLICT OF INTEREST STATEMENT

The authors declare no conflicts of interest.

## Supporting information


Appendix S1.

Appendix S2.

Appendix S3.

Appendix S4.

## Data Availability

Code (DeFilippo, 2026) is available in Figshare at https://doi.org/10.6084/m9.figshare.29963606.
