# Supplementary material for: Shifting stage‐specific constraints on productivity shape recovery potential for Yukon River Chinook salmon
Source: Ecol Appl. 2026 Apr 8;36(3):e70229. doi: 10.1002/eap.70229 (PMC13058899; doi:10.1002/eap.70229)
Supplement: Supplementary file 1 — Appendix S1. [file EAP-36-e70229-s004.pdf]

Shifting stage-specific constraints on productivity shape recovery potential for Yukon River Chinook salmon

Lukas B. DeFilippo, Kathrine G. Howard, Curry J. Cunningham, Robert M. Suryan, Patrick D. Barry, James Murphy, Wesley A. Larson

Ecological Applications

Appendix S1: Supporting information

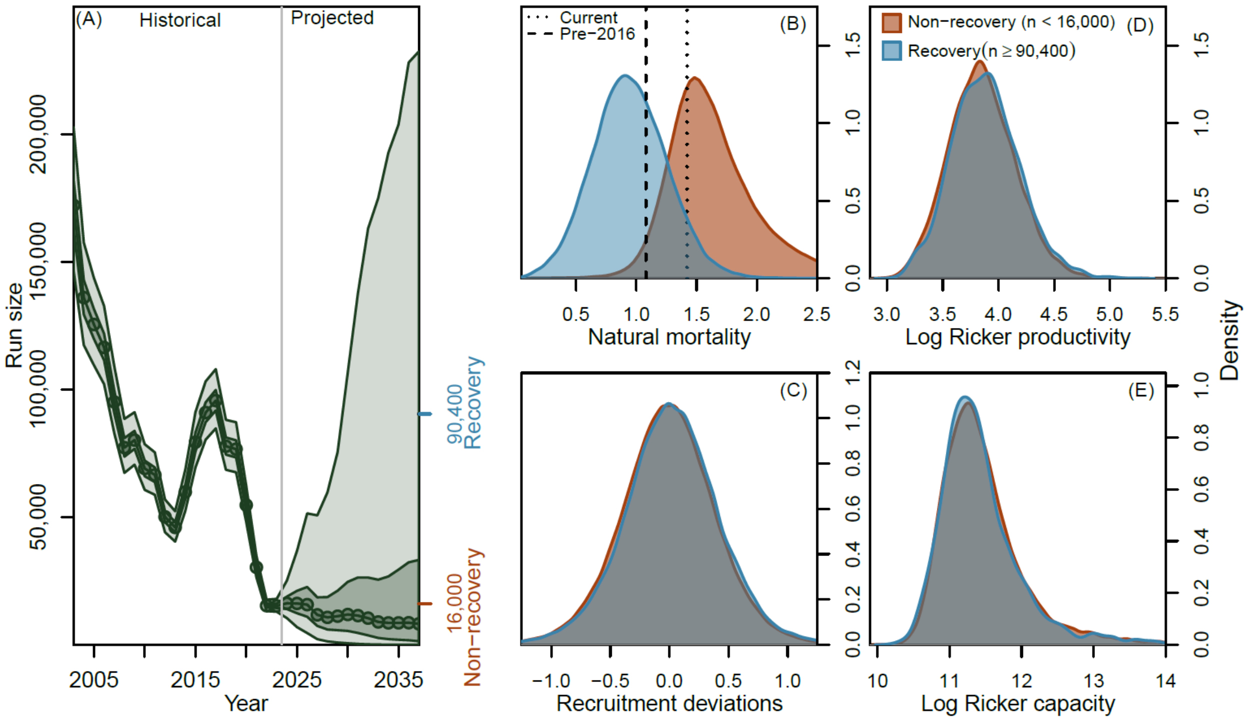

Figure S1. 14-year forward projection outcomes. Details of this figure are identical to those of Figure 7 (main text), except that projections depicted here were carried out over 14 (instead of 7) years. The 90th percentile of the final (2037) run size distribution (90,400 fish) is used to define the 'recovery' parameter space here.

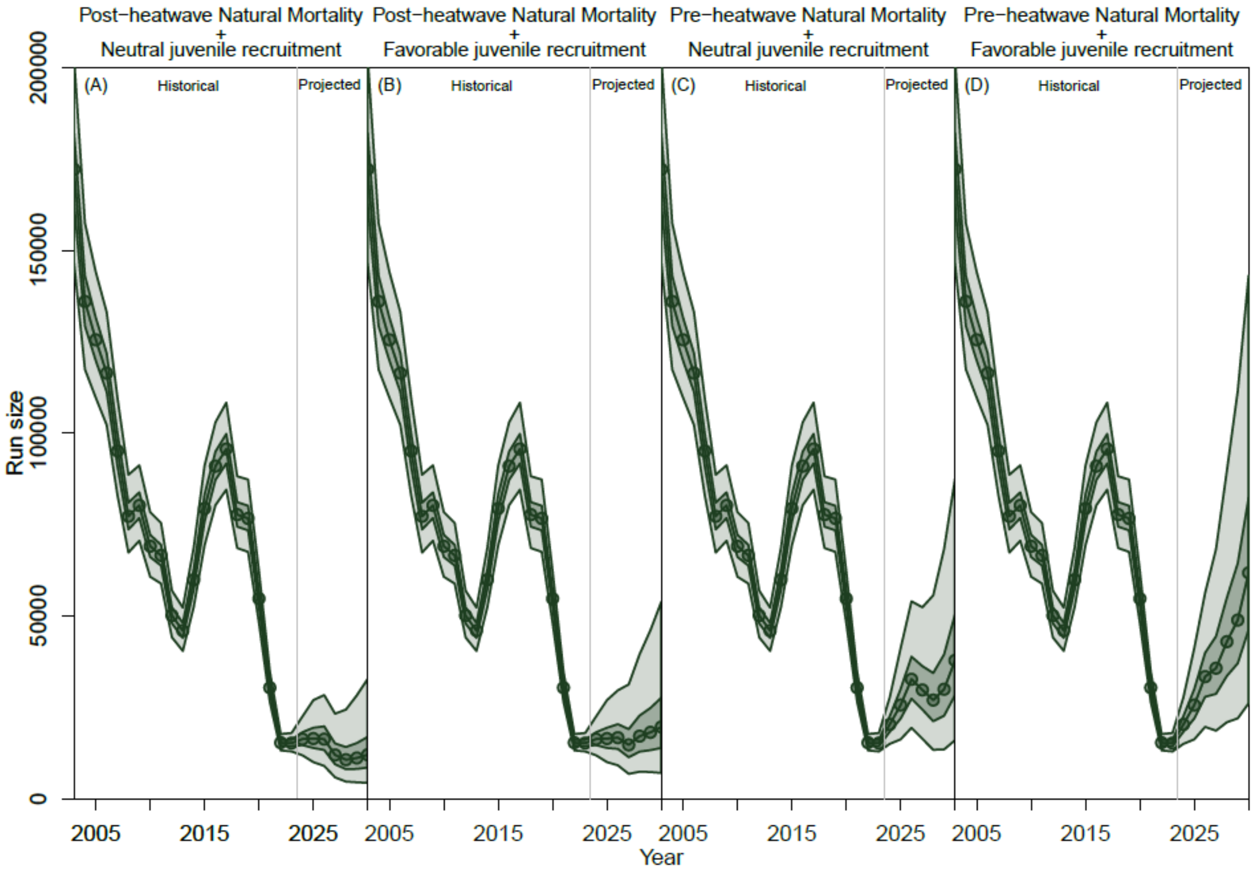

Figure S2. Model projection outcomes under fixed juvenile recruitment errors and post-juvenile natural mortality rates. Unlike the model projections described in the main text and depicted in Figure 7 (main text) where new recruitment deviations (ε_R) and post-juvenile natural mortality rates (M) during the projection window were simulated conditional on their estimated distributions from the fitted model, in the projections depicted here M_y and ε_{R_y} were fixed at pre-specified values in all years to explicitly explore the interactions between them in shaping recovery outcomes. We explored scenarios of consistently 'favorable juvenile recruitment' (*i.e.*, ε_{R_y} fixed at their 95th percentile value in all years from 2024 to 2030) versus 'neutral recruitment' (ε_{R_y} fixed at zero in all years from 2024 to 2030). We also explored post-juvenile natural mortality rates fixed at the median pre-heatwave (pre-2016) mortality level (prior to the increase in M from 2016 to 2023) in all years from 2024 to 2030, and fixed at the most recent (2023), post-heatwave median value from 2024 to 2030. Simulations were executed across all possible combinations of these recruitment and natural mortality scenarios. Panel A depicts the outcomes of projections executed with neutral recruitment and post-heatwave/recent (2023) natural mortality rates across the projection window, while panel B depicts projections with consistently favorable juvenile recruitment and post-heatwave/recent (2023) natural mortality. Panel C depicts projection outcomes with neutral juvenile recruitment errors and pre-heatwave (pre-2016) natural mortality, while panel D depicts projection outcomes under both favorable juvenile recruitment and pre-heatwave natural mortality.

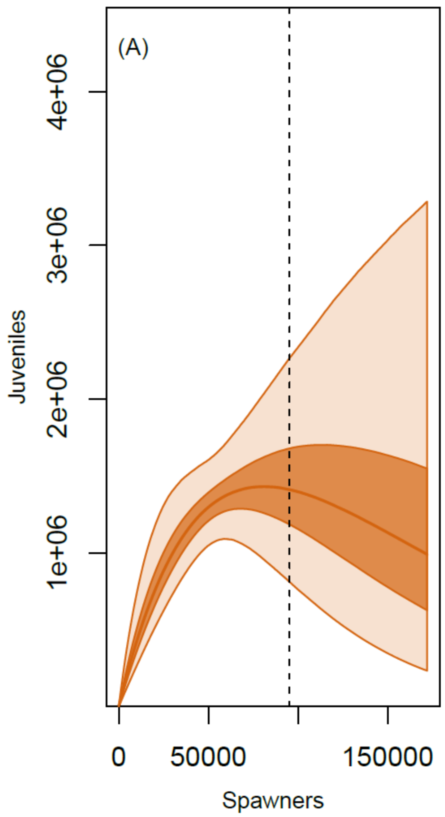

Figure S3. Estimated Ricker spawner-to-juvenile recruitment curve across the range of escapement sizes that occurred in the zero-harvest retrospective simulations. The maximum observed spawner escapement (the bounds of the x axis in Figure 3A, main text) is depicted as a dashed vertical line here. The upper x axis bound here represents the upper range of escapement abundances that occurred in the zero-harvest retrospective simulations. Note the greater degree of overcompensation for the range of escapement sizes occurring in the zero-harvest simulations shown here compared to the fitted model (Figure 3A, main text).

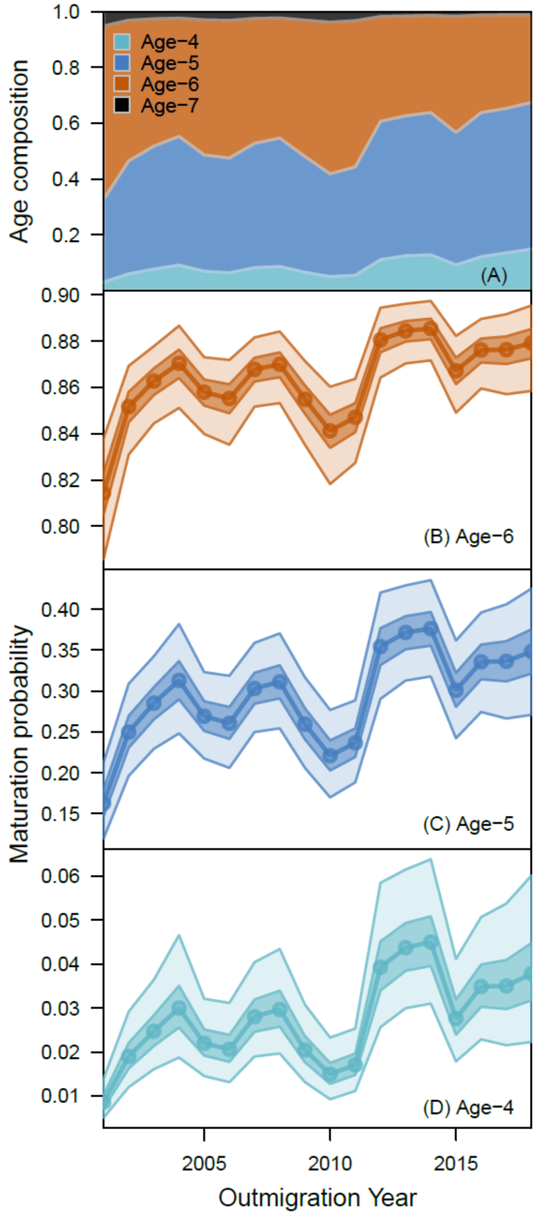

Figure S4. Age composition and maturation patterns. Panel A shows the median estimated age composition of cohorts by outmigration year, with the proportion of fish maturing at age-4, age-5, age-6, and age-7. Panels B-D show the estimated maturation probabilities-at-age over time ($\theta_{y_c,a}$) for each cohort by outmigration year as governed by the time-varying logistic maturation term (λ_{y_c}), with median values shown as filled circles and solid lines, and 50% and 95% credible intervals shown as dark and light shaded boundaries, respectively. While maturation probabilities-at-age were estimated for each juvenile cohort that contributed to observed returns across our study period, maturation estimates for incomplete cohorts (*i.e.*, those for which only a portion of possible age classes were observed in the mature population from 2003 to 2023) were considered less reliable. For instance, to model the proportion of age-7 fish in the 2003 run, an estimate of λ_{y_c} for the 1998 cohort of age-2 juveniles was necessary. However, no other age classes from the 1998 cohort were observed in the mature population during our study period, and thus $\lambda_{y_c=1998}$ is weakly informed. As such, only the age composition/maturation probabilities from complete cohorts are shown here, which consist of the 2001-2018 juvenile year classes.
