# Supplementary material for: Shifting stage‐specific constraints on productivity shape recovery potential for Yukon River Chinook salmon
Source: Ecol Appl. 2026 Apr 8;36(3):e70229. doi: 10.1002/eap.70229 (PMC13058899; doi:10.1002/eap.70229)
Supplement: Supplementary file 2 — Appendix S2. [file EAP-36-e70229-s003.pdf]

Shifting stage-specific constraints on productivity shape recovery potential for Yukon River Chinook salmon

Lukas B. DeFilippo, Kathrine G. Howard, Curry J. Cunningham, Robert M. Suryan, Patrick D. Barry, James Murphy, Wesley A. Larson

Ecological Applications

Appendix S2: Model fits to observed data

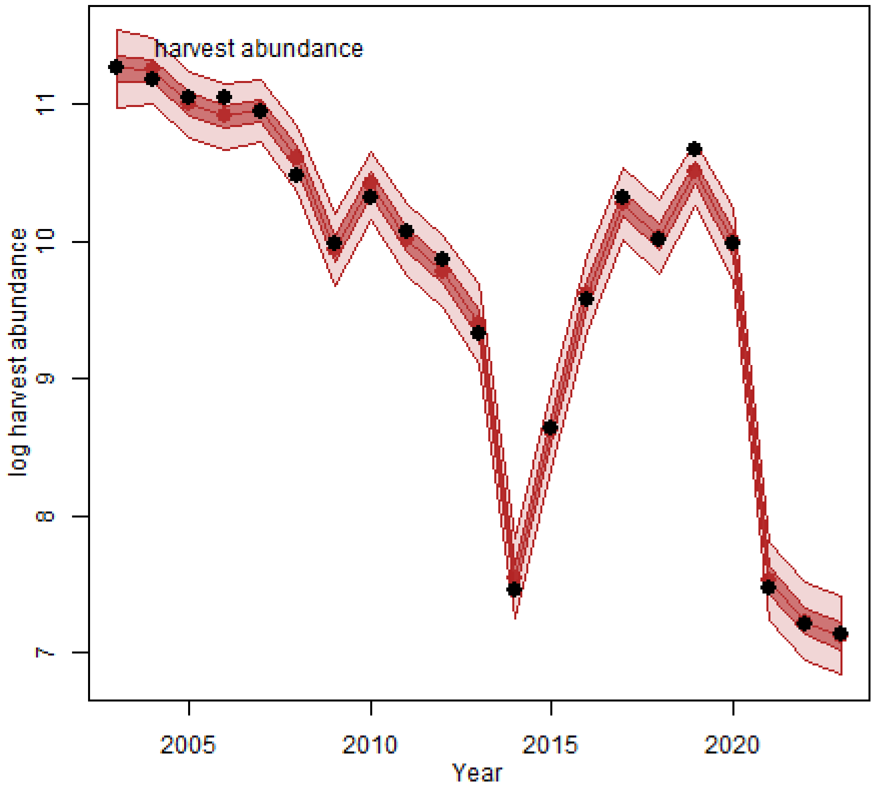

Figure S1. Model fits to harvest abundance information. Model estimates are shown in red, with median posterior estimates indicated by solid lines and filled circles, and 50% and 95% credible intervals indicated by dark and light shaded boundaries respectively. Harvest abundance data (output from the Yukon River Chinook salmon run reconstruction) are shown as filled black circles.

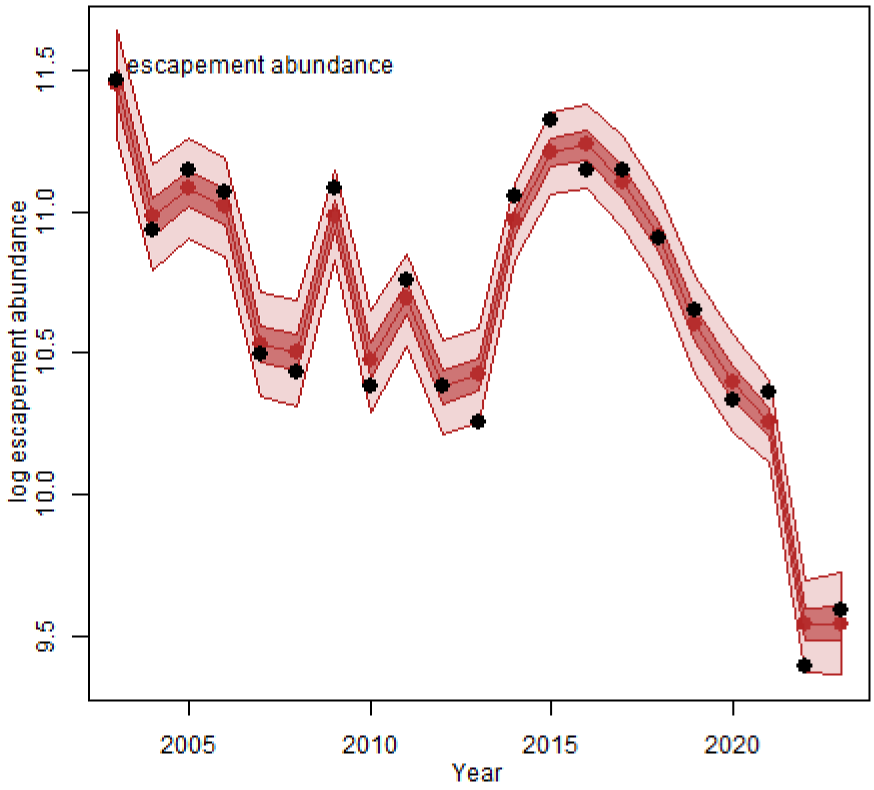

Figure S2. Model fits to escapement abundance information. Model estimates are shown in red, with median posterior estimates indicated by solid lines and filled circles, and 50% and 95% credible intervals indicated by dark and light shaded boundaries respectively. Escapement abundance data (output from the Yukon River Chinook salmon run reconstruction) are shown as filled black circles.

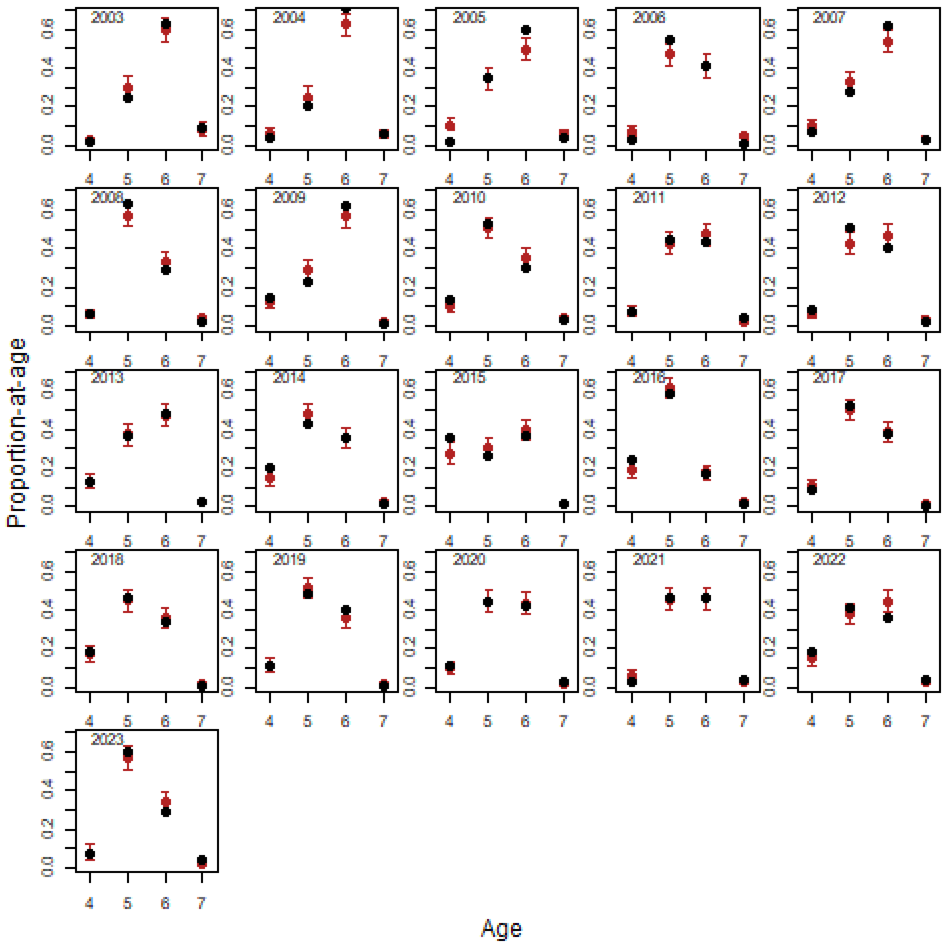

Figure S3. Model fits to harvest age composition information. Model estimates are shown in red, with median posterior estimates indicated by filled circles, and 50% and 95% credible intervals indicated by thick and thin lines respectively. Harvest age composition data (output from the run reconstruction) are shown as filled black circles. Individual panels show the proportions of each age class present in the mature population (4-7) in a given year.

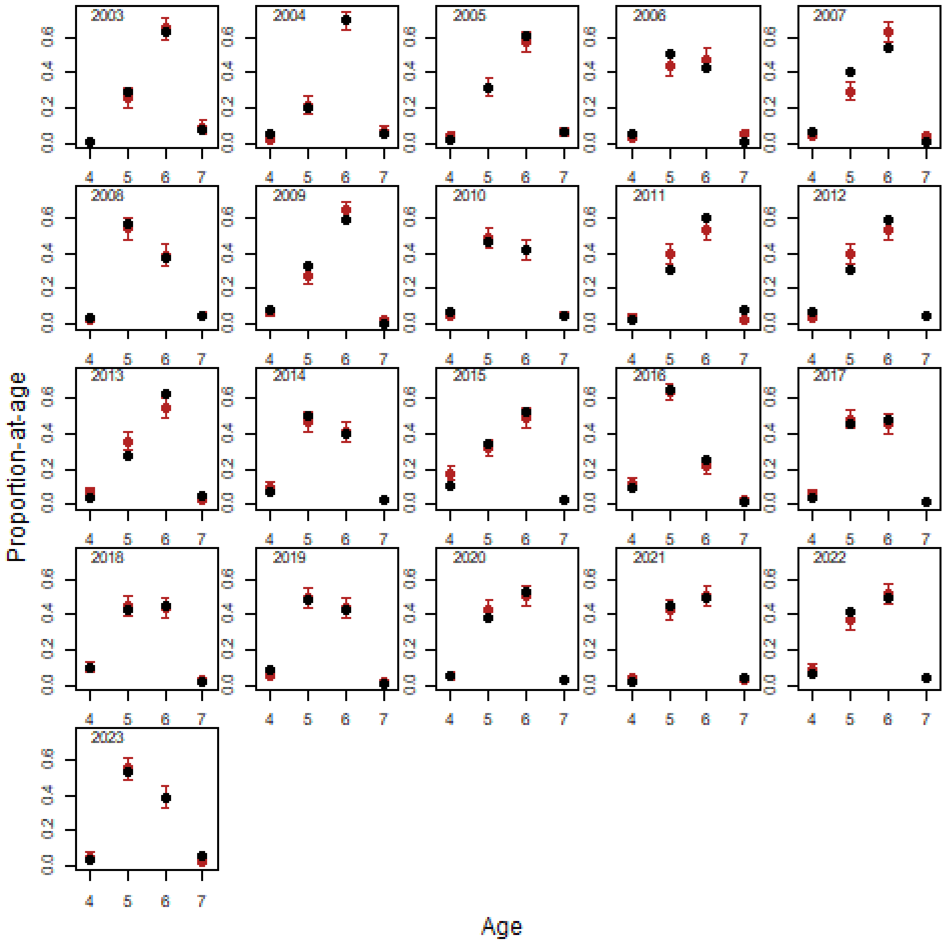

Figure S4. Model fits to escapement age composition information. Model estimates are shown in red, with median posterior estimates indicated by filled circles, and 50% and 95% credible intervals indicated by thick and thin lines respectively. Escapement age composition data (output from the run reconstruction) are shown as filled black circles. Individual panels show the proportions of each age class present in the mature population (4-7) in a given year.

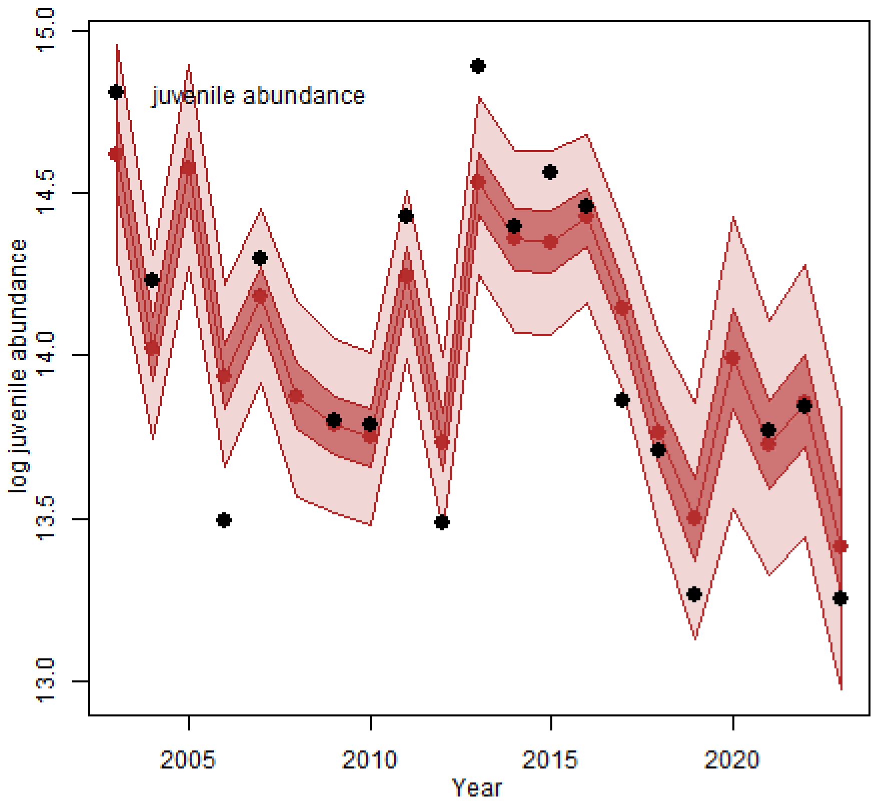

Figure S5. Model fits to juvenile abundance information. Model estimates are shown in red, with median posterior estimates indicated by solid lines and filled circles, and 50% and 95% credible intervals indicated by dark and light shaded boundaries respectively. The abundance data on of upper (Canada-origin) Yukon River Chinook salmon from the NBEST survey is shown as filled black circles.

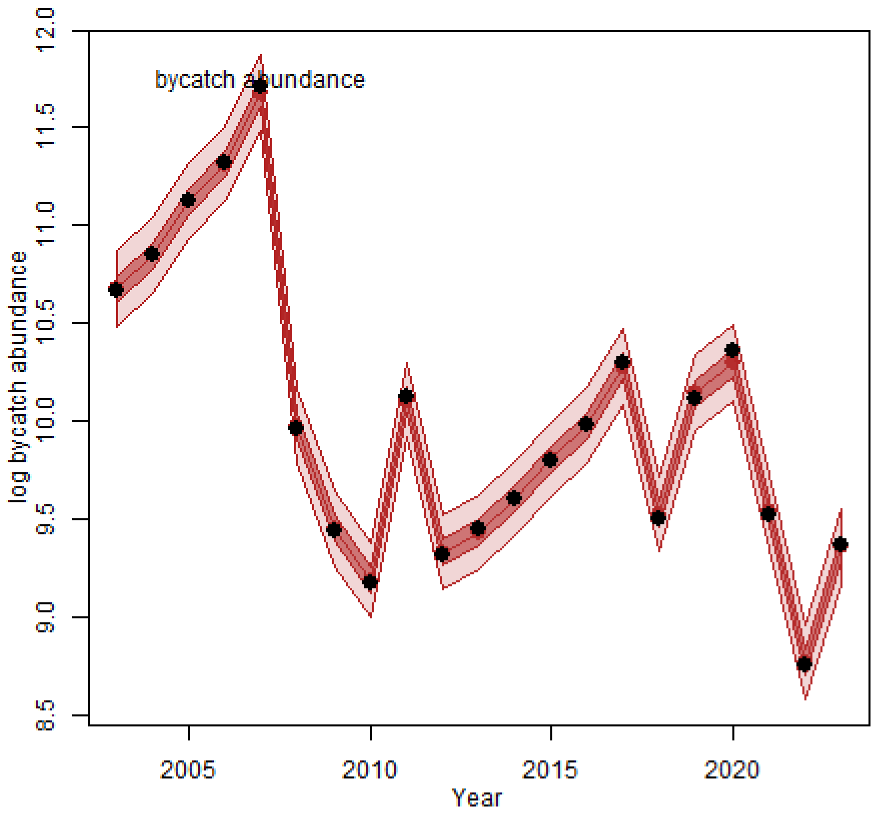

Figure S6. Model fits to bycatch abundance information. Model estimates are shown in red, with median posterior estimates indicated by solid lines and filled circles, and 50% and 95% credible intervals indicated by dark and light shaded boundaries respectively. The total abundance of Chinook salmon bycatch (data) is shown as filled black circles.

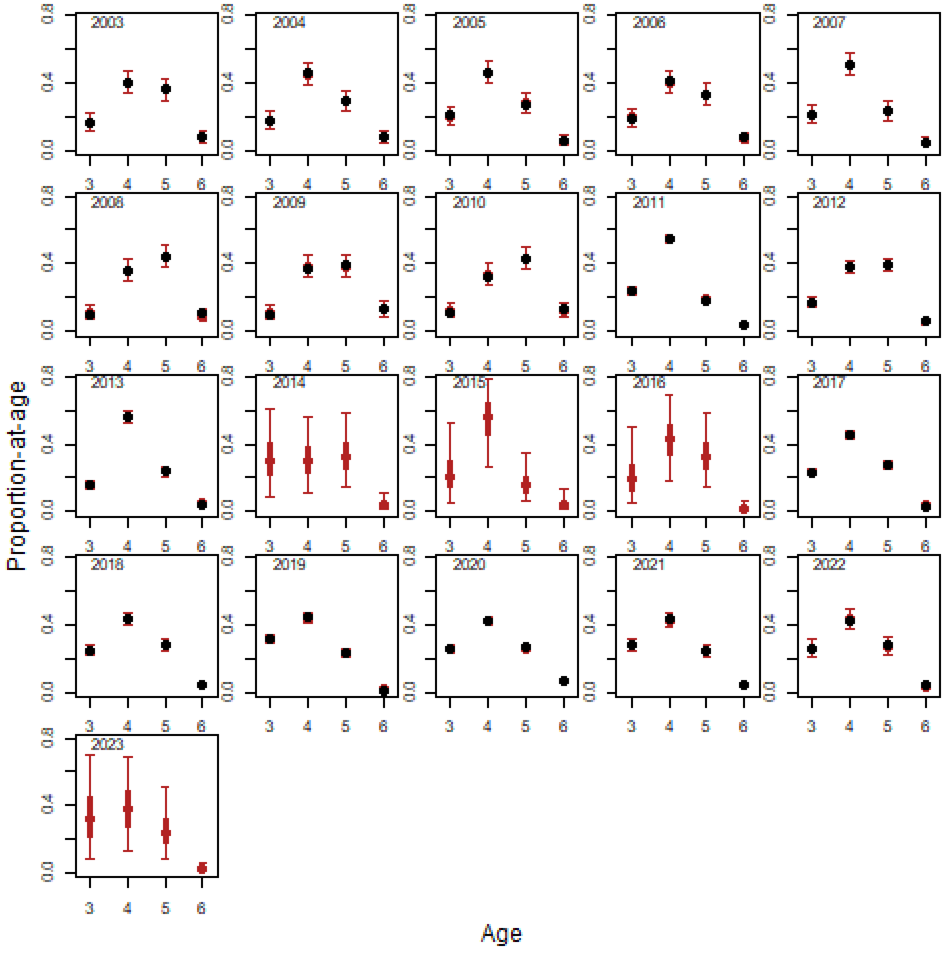

Figure S7. Model fits to Chinook salmon bycatch age composition information. Model estimates are shown in red, with median posterior estimates indicated by filled circles, and 50% and 95% credible intervals indicated by thick and thin lines respectively. Age composition data for the aggregate Chinook salmon bycatch in the EBS pollock fishery is shown as black circles. Individual panels show the proportions of each age class present in bycatch across ages (3-6) by year.

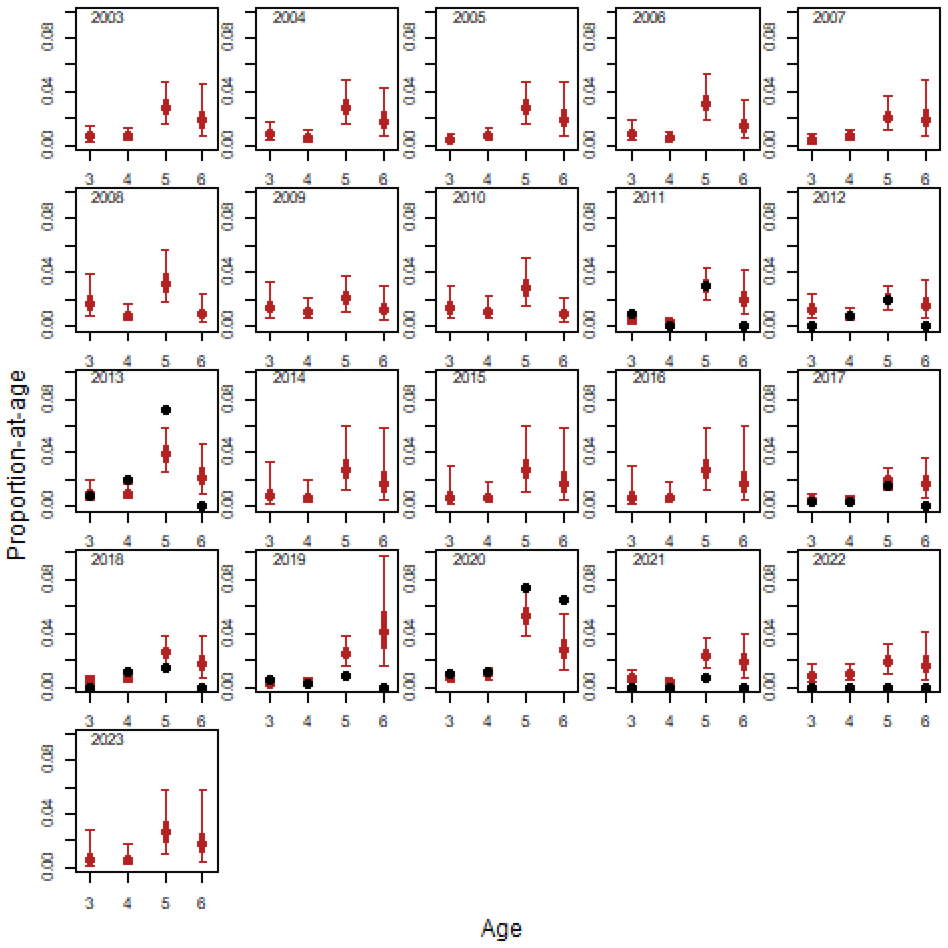

Figure S8. Model fits to Chinook salmon bycatch stock composition-at-age information. Model estimates are shown in red, with median posterior estimates indicated by filled circles, and 50% and 95% credible intervals indicated by thick and thin lines respectively. The proportions-at-age of the bycatch genetically assigned to the upper Yukon reporting group (data) are shown as black circles. Note that the lack of fit in some instances is likely due to a combination of two factors. First, the hierarchical distribution of bycatch stock composition-at-age necessarily induces some degree of bias by shrinking annual estimates towards the among-year mean. Second, the sample sizes for the bycatch stock composition-at-age were often small (n < 50-100) such that even if the stock composition data indicated that 0% of a given age class belonged to the upper Yukon reporting group, the binomial probability that the true stock composition was greater than zero was still appreciable. As such, we do not consider any apparent biases in model estimates of bycatch stock composition-at-age relative to the observed data to represent a cause for concern, but rather reflects appropriate probabilistic weighting of the available information.
