# Supplementary material for: Shifting stage‐specific constraints on productivity shape recovery potential for Yukon River Chinook salmon
Source: Ecol Appl. 2026 Apr 8;36(3):e70229. doi: 10.1002/eap.70229 (PMC13058899; doi:10.1002/eap.70229)
Supplement: Supplementary file 3 — Appendix S3. [file EAP-36-e70229-s001.pdf]

Shifting stage-specific constraints on productivity shape recovery potential for Yukon River Chinook salmon

Lukas B. DeFilippo, Kathrine G. Howard, Curry J. Cunningham, Robert M. Suryan, Patrick D. Barry,

James Murphy, Wesley A. Larson

Ecological Applications

Appendix S3. Posterior predictive checks.

In all posterior predictive checks, simulated posterior predictive distributions for each data type were generated from the fitted model and compared to the observed data.

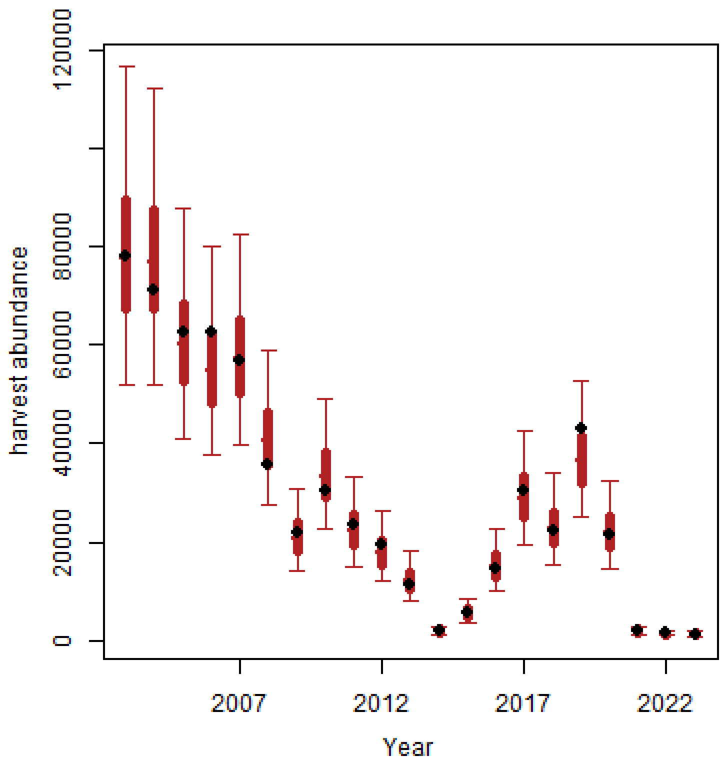

Figure S1. Posterior predictive check of harvest abundance data. Posterior predictive distributions from the IPM are shown in red, with 50% and 95% posterior predictive distributions shown as thick and thin lines respectively. Harvest abundance data from the run reconstruction are shown as black circles.

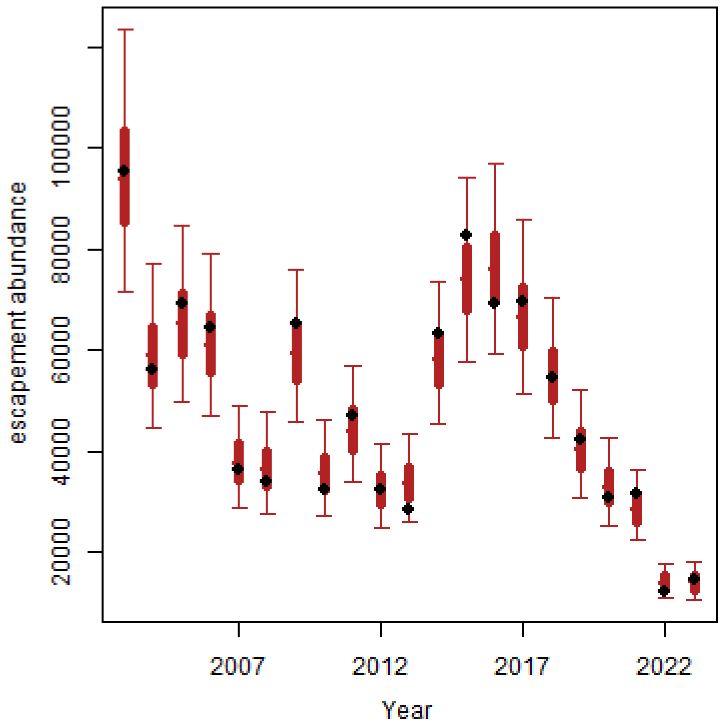

Figure S2. Posterior predictive check of escapement abundance data. Posterior predictive distributions from the IPM are shown in red, with 50% and 95% posterior predictive distributions shown as thick and thin lines respectively. Escapement abundance data from the run reconstruction are shown as black circles.

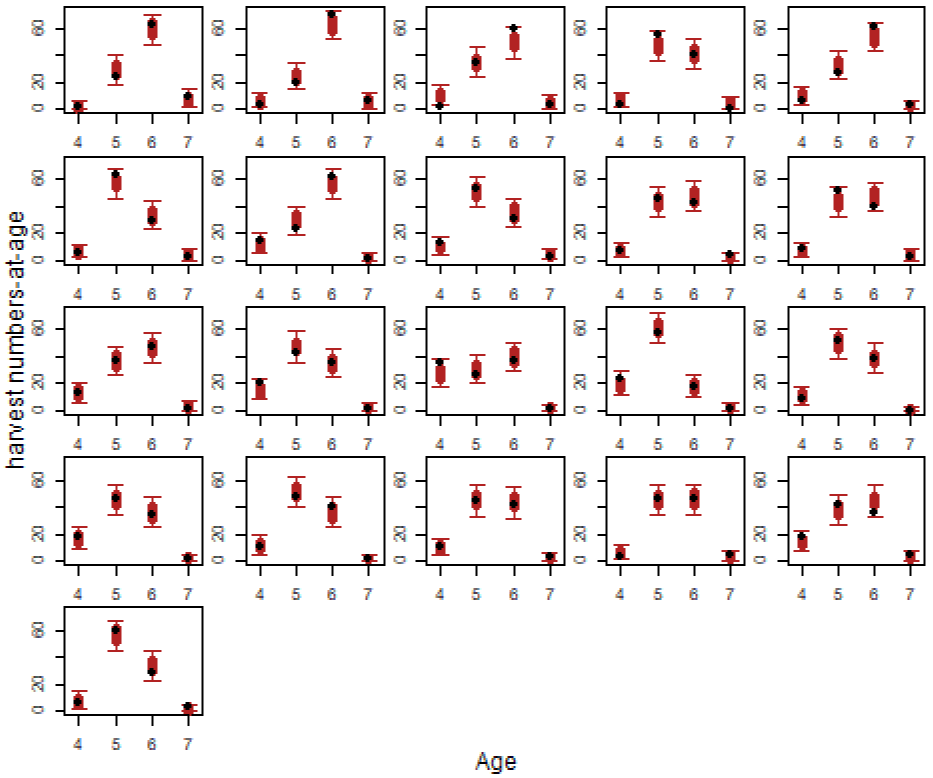

Figure S3. Posterior predictive check of harvest age composition data. Posterior predictive distributions are shown in red with 50% and 95% posterior predictive intervals indicated by thick and thin lines respectively. Harvest age composition samples-at-age from the run reconstruction (data) are shown as filled black circles. Each panel represents a given year of data.

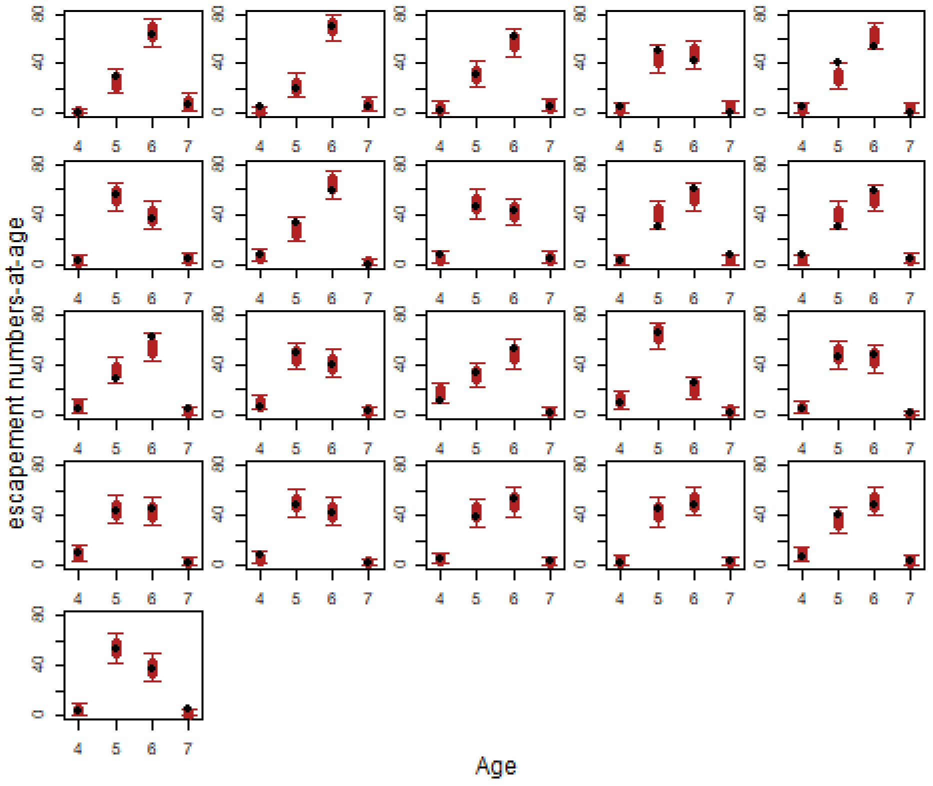

Figure S4. Posterior predictive check of escapement age composition data. Posterior predictive distributions are shown in red with 50% and 95% posterior predictive intervals indicated by thick and thin lines respectively. Escapement age composition samples-at-age from the run reconstruction (data) are shown as filled black circles. Each panel represents a given year of data.

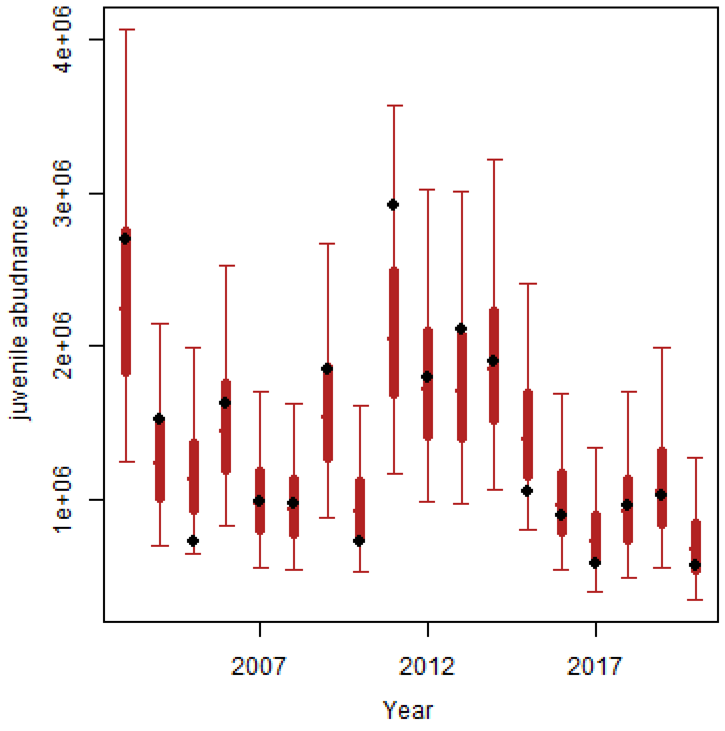

Figure S5. Posterior predictive check of juvenile abundance data. Posterior predictive distributions from the IPM are shown in red, with 50% and 95% posterior predictive distributions shown as thick and thin lines respectively. Juvenile abundance indices from the NBEST survey are shown as black circles.

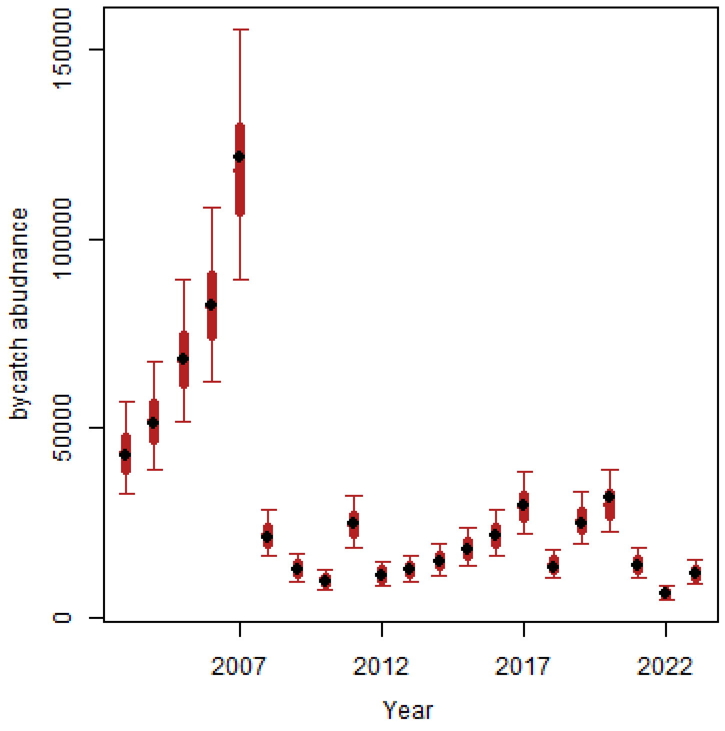

Figure S6. Posterior predictive check of Chinook salmon bycatch data from the EBS pollock fishery. Posterior predictive distributions from the IPM are shown in red, with 50% and 95% posterior predictive distributions shown as thick and thin lines respectively. Aggregate Chinook salmon bycatch abundance data are shown as black circles.

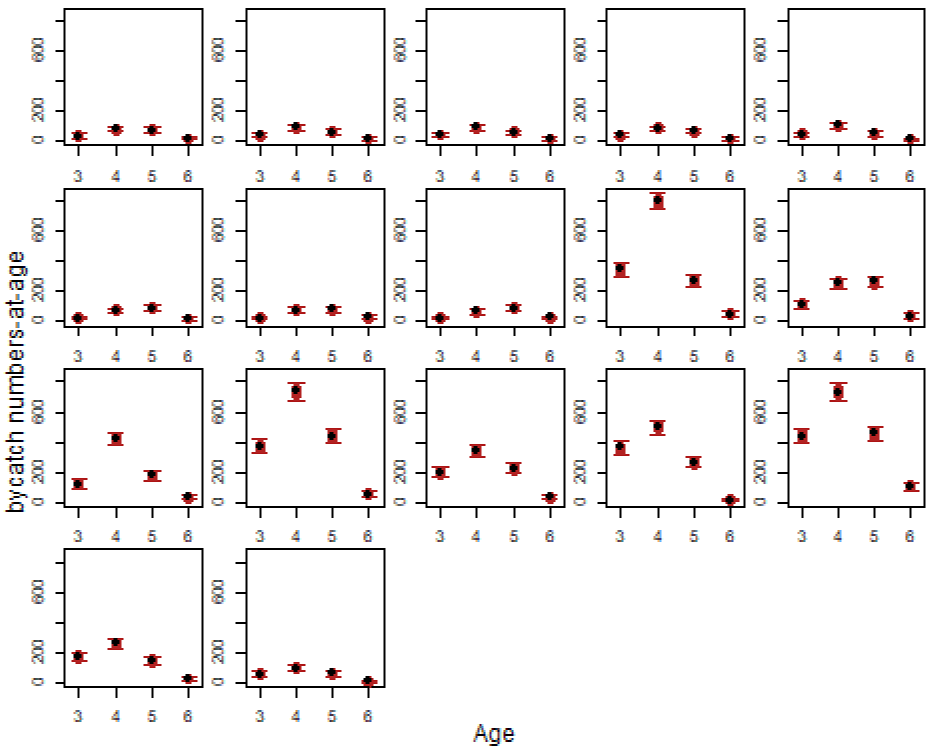

Figure S7. Posterior predictive check of bycatch age composition data. Posterior predictive distributions are shown in red with 50% and 95% posterior predictive intervals indicated by thick and thin lines respectively. Chinook salmon age composition samples from the EBS pollock fishery (data) are shown as filled black circles. Each panel represents a given year of data.

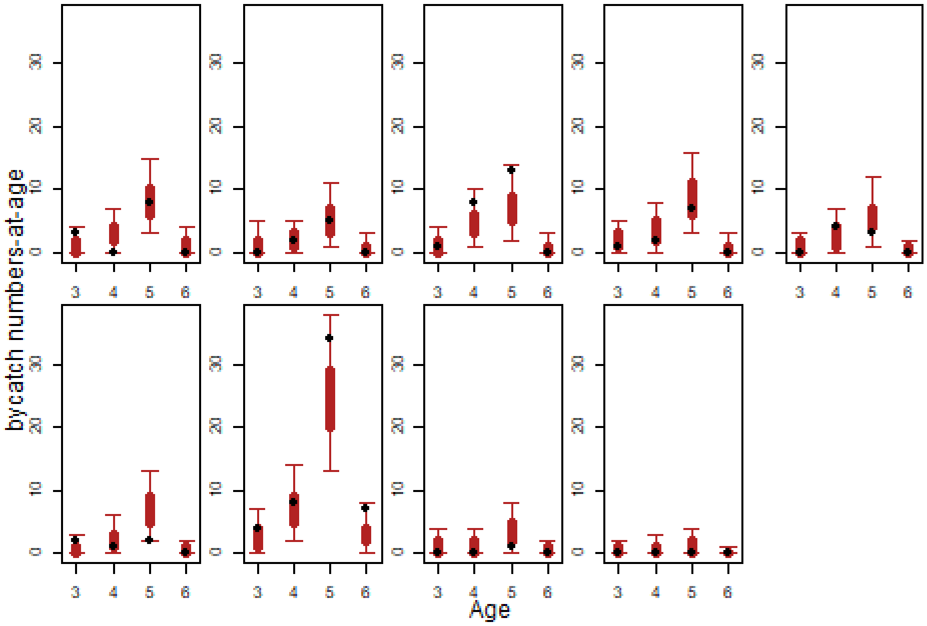

Figure S8. Posterior predictive check of bycatch stock composition-at-age data. Posterior predictive distributions are shown in red with 50% and 95% posterior predictive intervals indicated by thick and thin lines respectively. Chinook salmon bycatch samples from the EBS pollock fishery genetically assigned to the upper Yukon reporting group (data) are shown as filled black circles. Each panel represents a given year of available data.
