# Supplementary material for: Shifting stage‐specific constraints on productivity shape recovery potential for Yukon River Chinook salmon
Source: Ecol Appl. 2026 Apr 8;36(3):e70229. doi: 10.1002/eap.70229 (PMC13058899; doi:10.1002/eap.70229)
Supplement: Supplementary file 4 — Appendix S4. [file EAP-36-e70229-s002.pdf]

Shifting stage-specific constraints on productivity shape recovery potential for Yukon River Chinook salmon

Lukas B. DeFilippo, Kathrine G. Howard, Curry J. Cunningham, Robert M. Suryan, Patrick D. Barry,

James Murphy, Wesley A. Larson

Ecological Applications

Appendix S4. Model fits to simulated data.

Population dynamics were simulated following the same structural form as the IPM, with parameter values drawn from estimated posterior values from the fitted model. Fake data were simulated from model predictions of true harvest abundance, escapement abundance, juvenile abundance, harvest age composition, escapement age composition, total bycatch abundance and age composition and age-specific bycatch stock composition with errors based on the assumed lognormal observation error terms (abundance data) and multinomial effective sample sizes (compositional data) specified in the fitted model. Years with missing data for a given data type in reality were also assumed to be missing data in the generation of fake data from the simulations. The IPM was then fitted to the simulated data with errors, and the model's ability to recover the true underlying parameters specified in the simulation was assessed to gauge model performance. For all figures in this appendix, the black dots represent the true values, and the red lines/shading represent the model estimates.

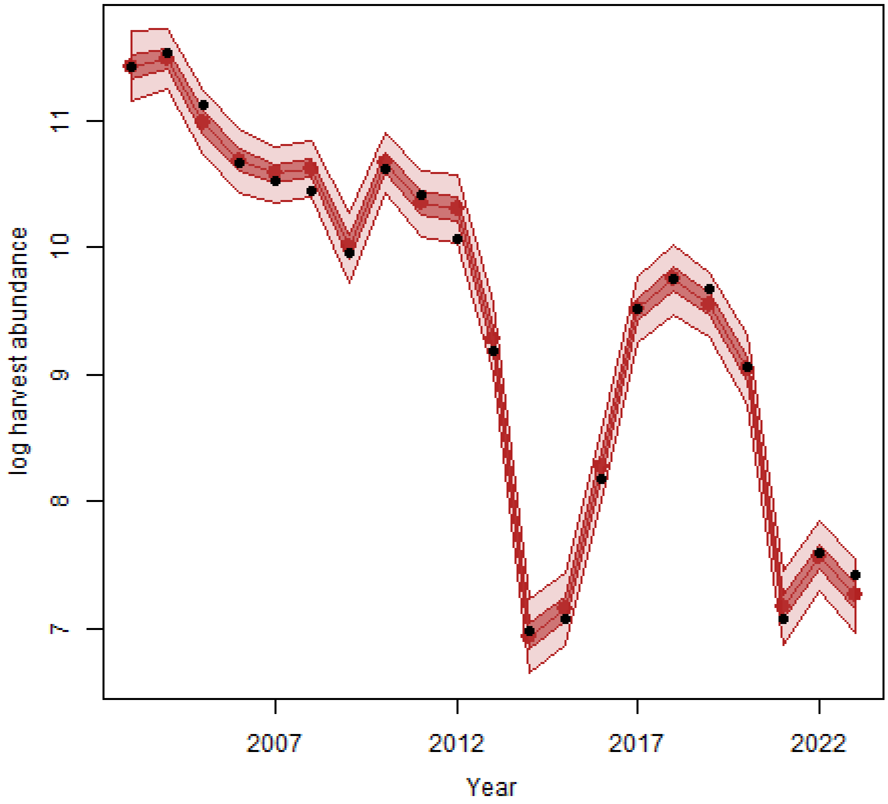

Figure S1. Model estimates of harvest abundance relative to true simulated values. The true harvest abundance specified in the simulation is shown in black, and model estimates based on fits to fake data simulated (with errors) are shown in red. The median model estimate is indicated by the circles and lines, and 50% and 95% credible intervals are indicated by dark and light shaded boundaries respectively.

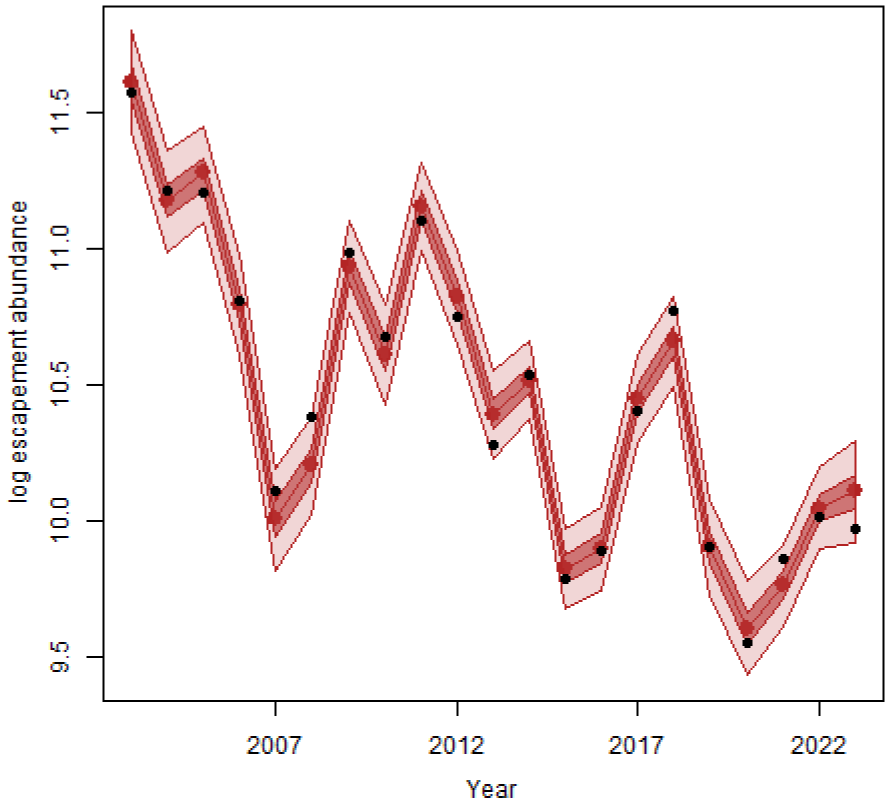

Figure S2. Model estimates of escapement abundance relative to true simulated values. The true escapement abundance specified in the simulation is shown in black, and model estimates based on fits to fake data simulated (with errors) are shown in red. The median model estimate is indicated by the circles and lines, and 50% and 95% credible intervals are indicated by dark and light shaded boundaries respectively.

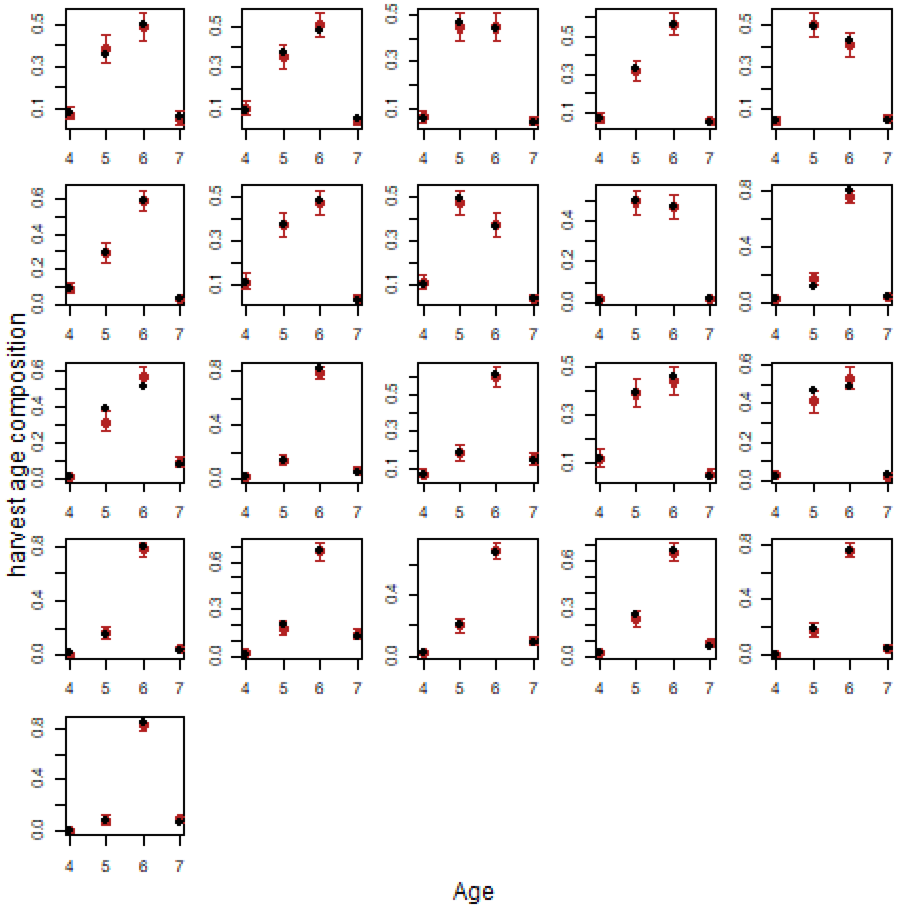

Figure S3. Model estimates of harvest age composition relative to true simulated values. The true harvest proportions-at-age specified in the simulation are shown in black, and model estimates based on fits to fake data simulated (with errors) are shown in red. The median model estimates are indicated by the circles, with 50% and 95% credible intervals indicated by thick and thin lines respectively. Each panel represents a given year of age composition estimates.

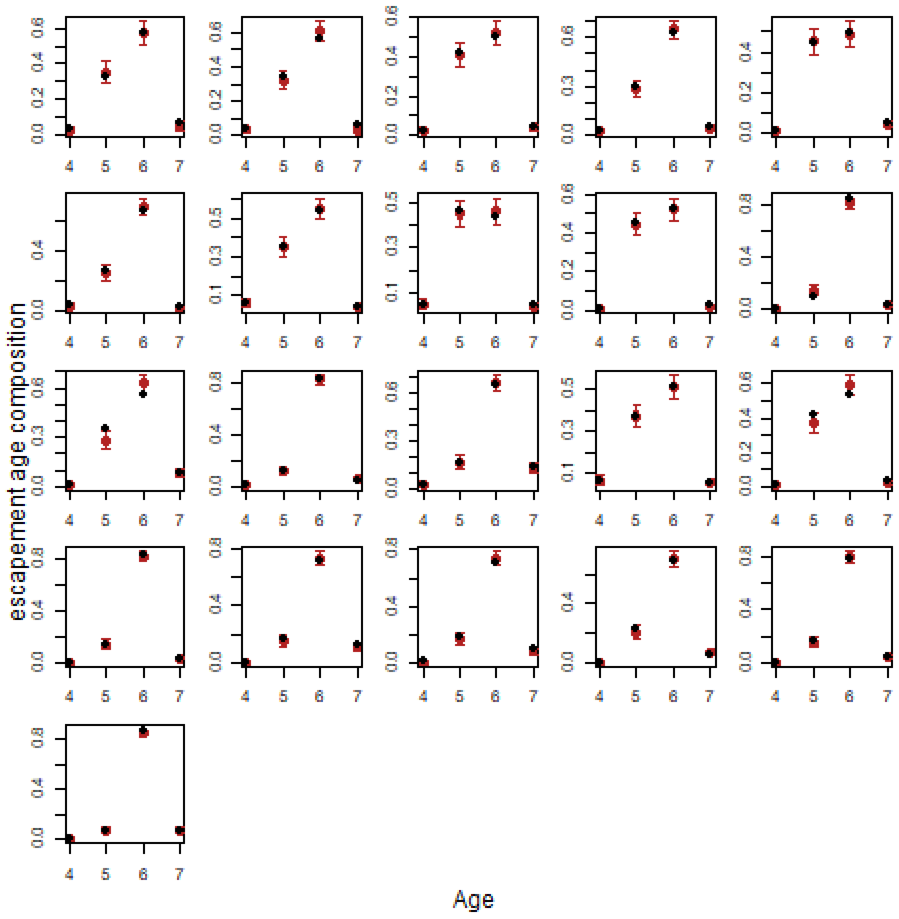

Figure S4. Model estimates of escapement age composition relative to true simulated values. The true escapement proportions-at-age specified in the simulation are shown in black, and model estimates based on fits to fake data simulated (with errors) are shown in red. The median model estimates are indicated by the circles, with 50% and 95% credible intervals indicated by thick and thin lines respectively. Each panel represents a given year of age composition estimates.

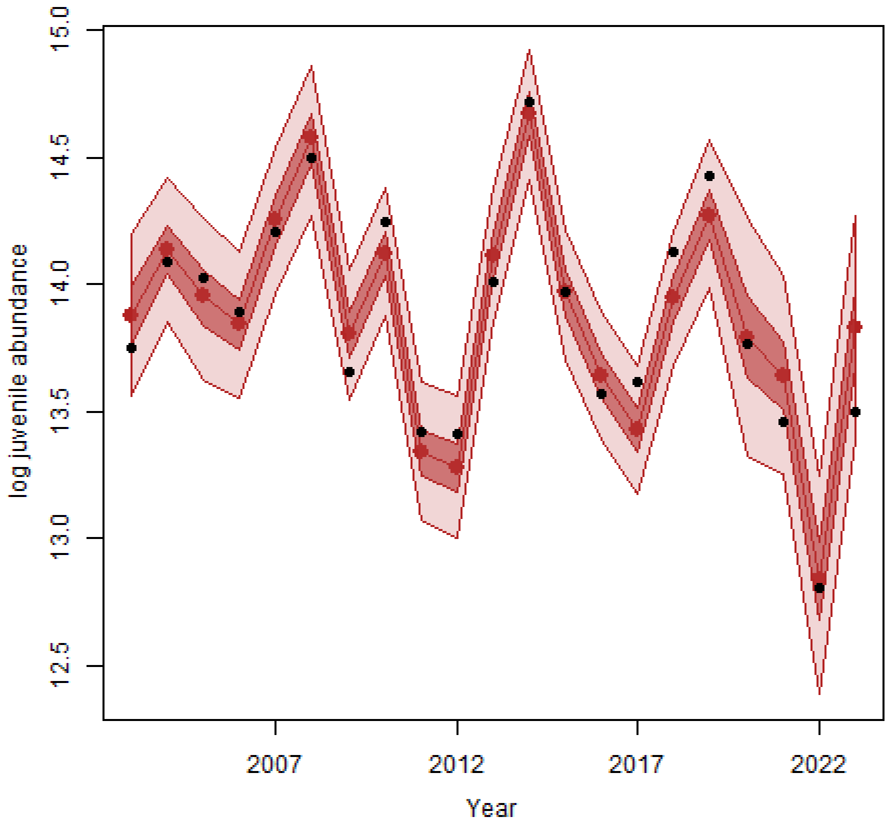

Figure S5. Model estimates of juvenile abundance relative to true simulated values. The true juvenile abundance specified in the simulation is shown in black, and model estimates based on fits to fake data simulated (with errors) are shown in red. The median model estimate is indicated by the circles and lines, and 50% and 95% credible intervals are indicated by dark and light shaded boundaries respectively.

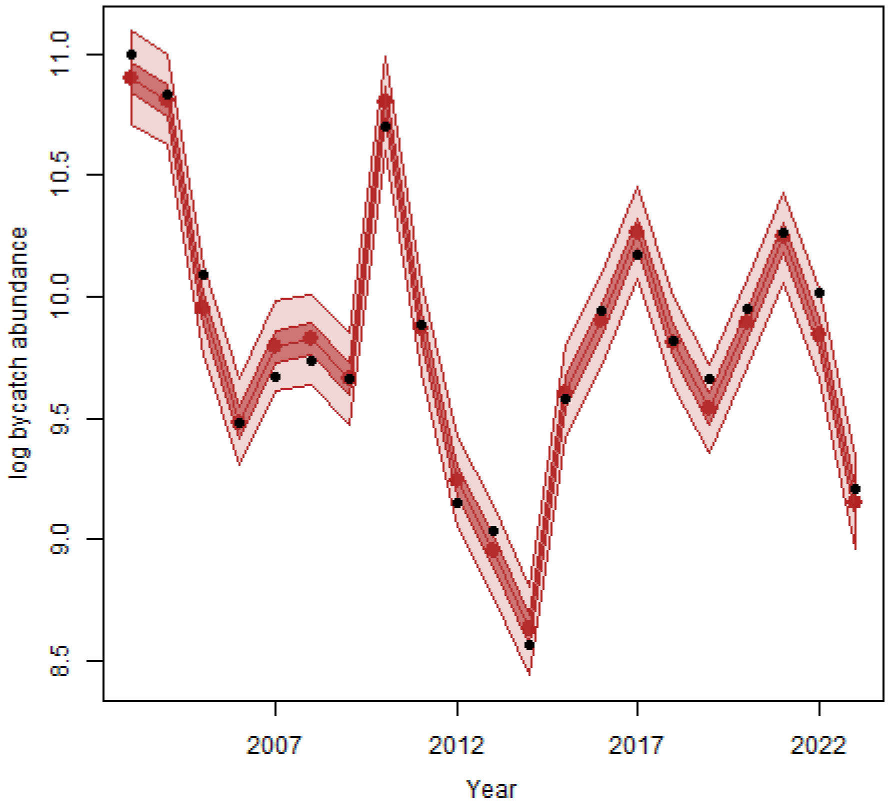

Figure S6. Model estimates of total bycatch abundance relative to true simulated values. The true bycatch abundance specified in the simulation is shown in black, and model estimates based on fits to fake data simulated (with errors) are shown in red. The median model estimate is indicated by the circles and lines, and 50% and 95% credible intervals are indicated by dark and light shaded boundaries respectively.

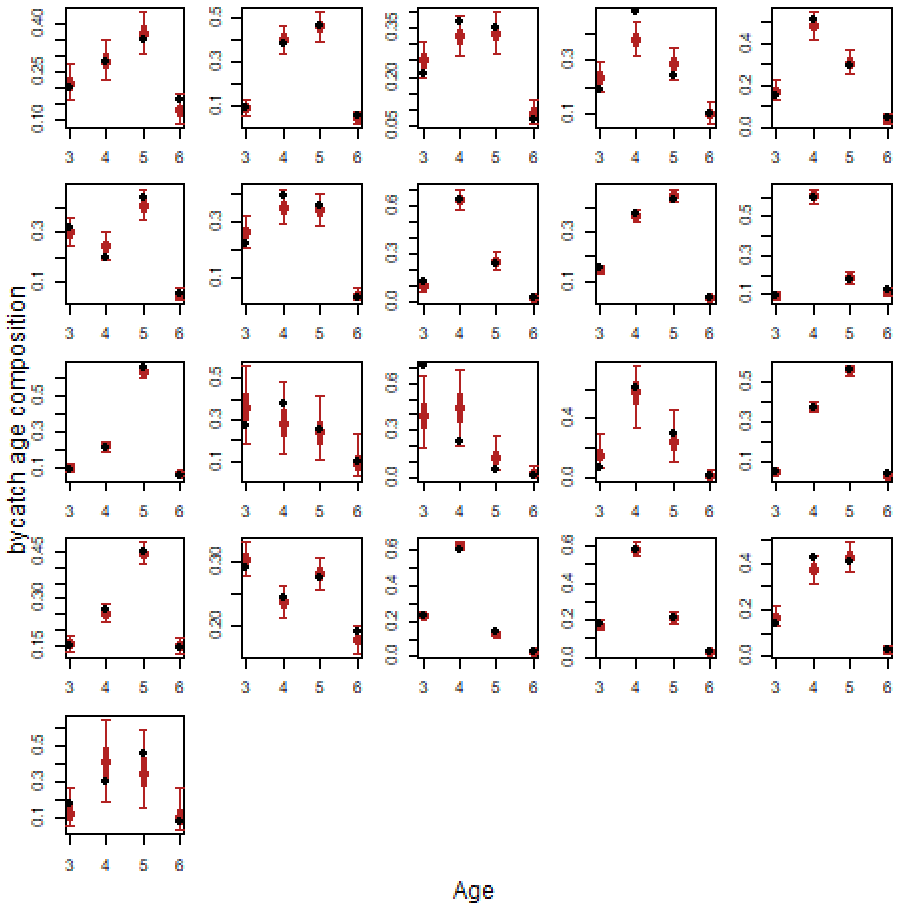

Figure S7. Model estimates of bycatch age composition relative to true simulated values. The true bycatch proportions-at-age specified in the simulation are shown in black, and model estimates based on fits to fake data simulated (with errors) are shown in red. The median model estimates are indicated by the circles, with 50% and 95% credible intervals indicated by thick and thin lines respectively. Each panel represents a given year of age composition estimates.

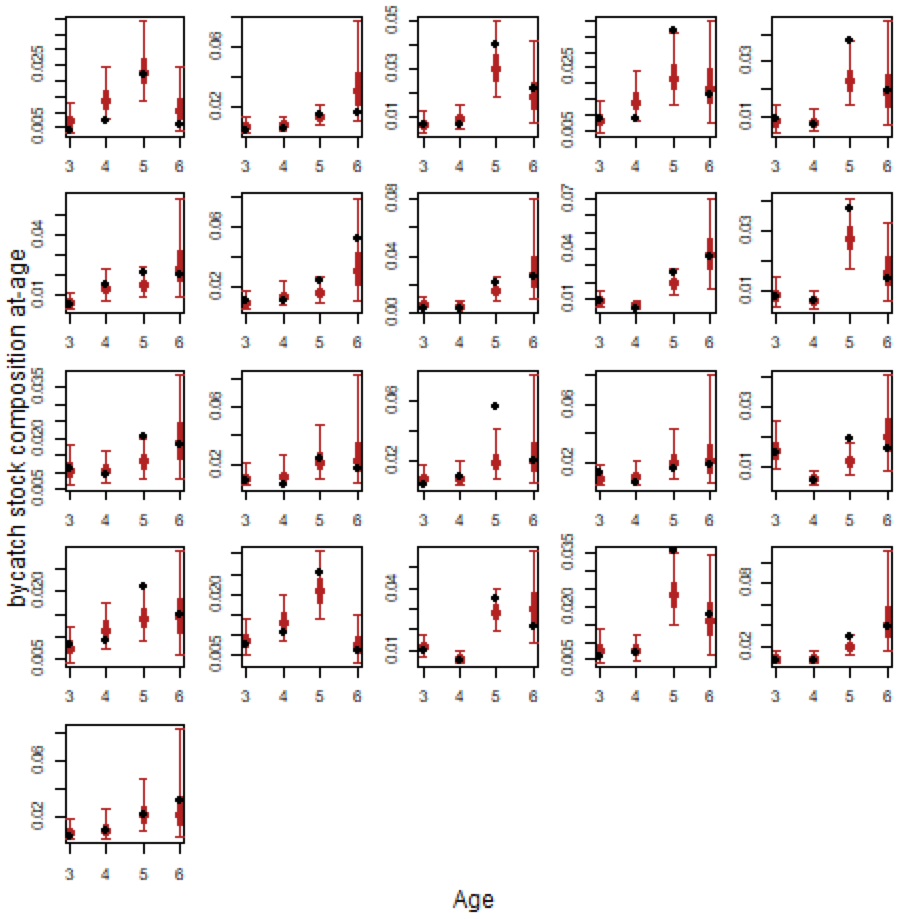

Figure S8. Model estimates of bycatch stock composition-at-age relative to true simulated values. The true bycatch proportions-at-age belonging to the upper (Canada-origin) Yukon stock specified in the simulation are shown in black, and model estimates based on fits to fake data simulated (with errors) are shown in red. The median model estimates are indicated by the circles, with 50% and 95% credible intervals indicated by thick and thin lines respectively. Each panel represents a given year of stock composition-at-age estimates.

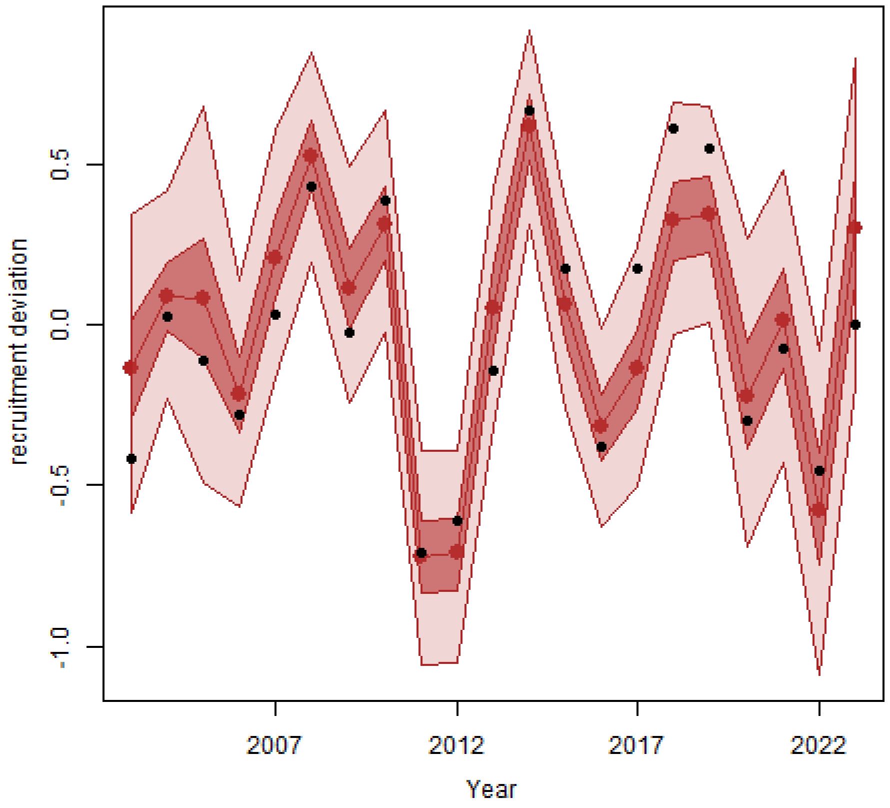

Figure S9. Model estimates of recruitment errors relative to true simulated values. The true recruitment errors specified in the simulation is shown in black, and model estimates based on fits to fake data simulated (with errors) are shown in red. The median model estimate is indicated by the circles and lines, and 50% and 95% credible intervals are indicated by dark and light shaded boundaries respectively.

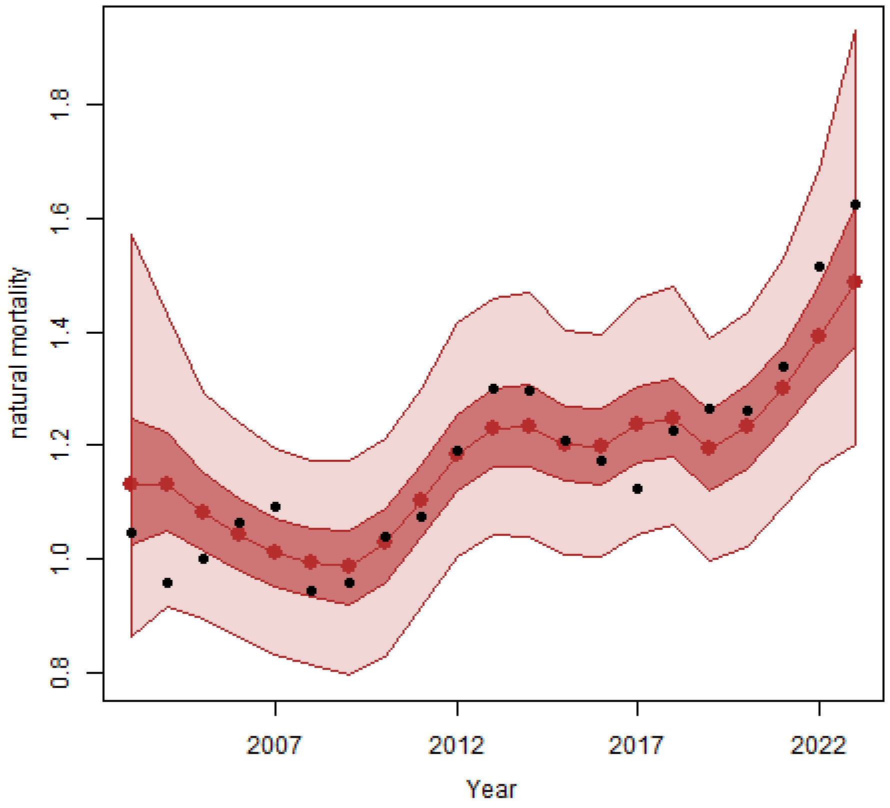

Figure S10. Model estimates of natural mortality relative to true simulated values. The true natural mortality values specified in the simulation is shown in black, and model estimates based on fits to fake data simulated (with errors) are shown in red. The median model estimate is indicated by the circles and lines, and 50% and 95% credible intervals are indicated by dark and light shaded boundaries respectively.

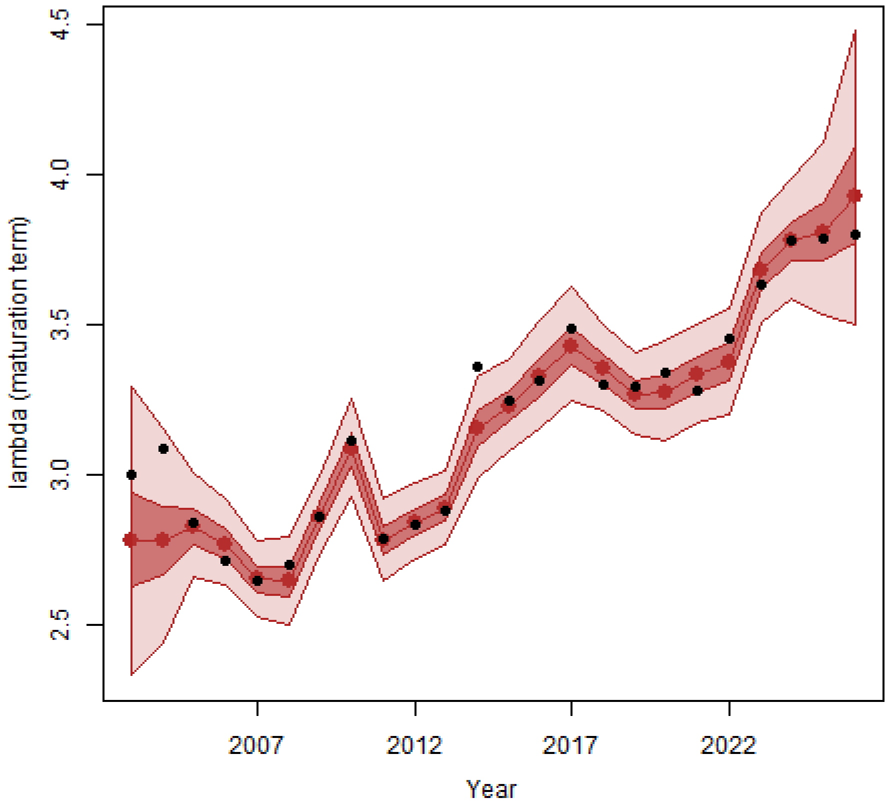

Figure S11. Model estimates of the maturation schedule term relative to true simulated values. The true maturation schedule parameter values specified in the simulation is shown in black, and model estimates based on fits to fake data simulated (with errors) are shown in red. The median model estimate is indicated by the circles and lines, and 50% and 95% credible intervals are indicated by dark and light shaded boundaries respectively.

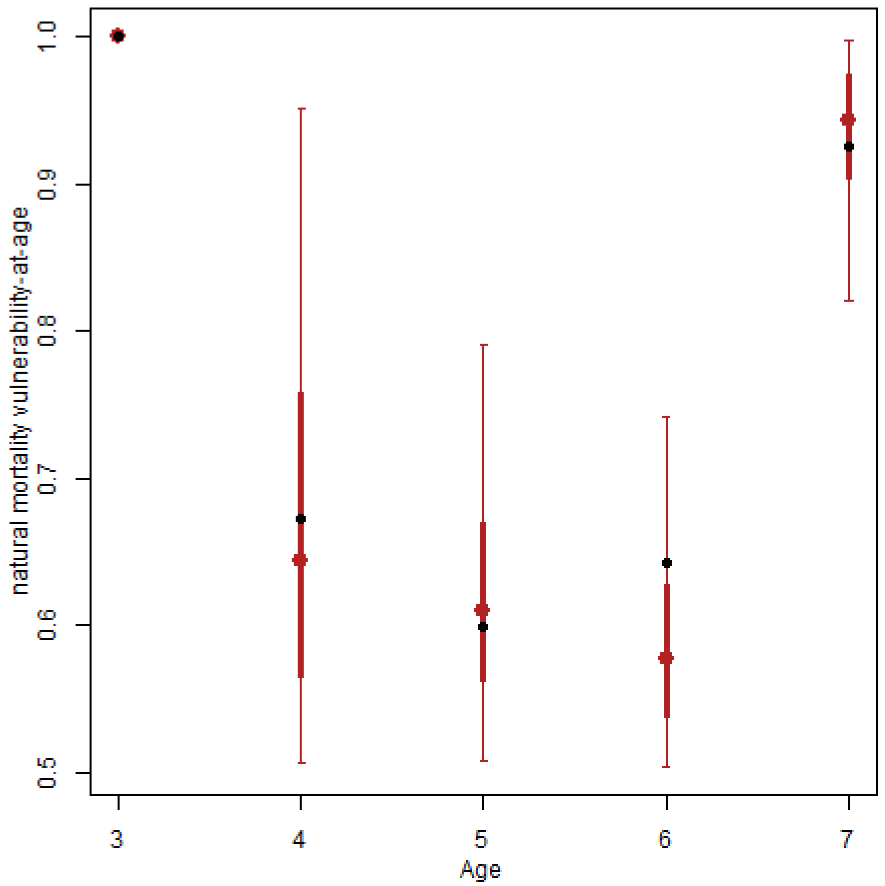

Figure S12. Model estimates of vulnerability-at-age to natural mortality relative to true simulated values. The true vulnerability-at-age to natural mortality values specified in the simulation are shown in black, and model estimates based on fits to fake data simulated (with errors) are shown in red. The median model estimates are indicated by the circles, with 50% and 95% credible intervals indicated by thick and thin lines respectively

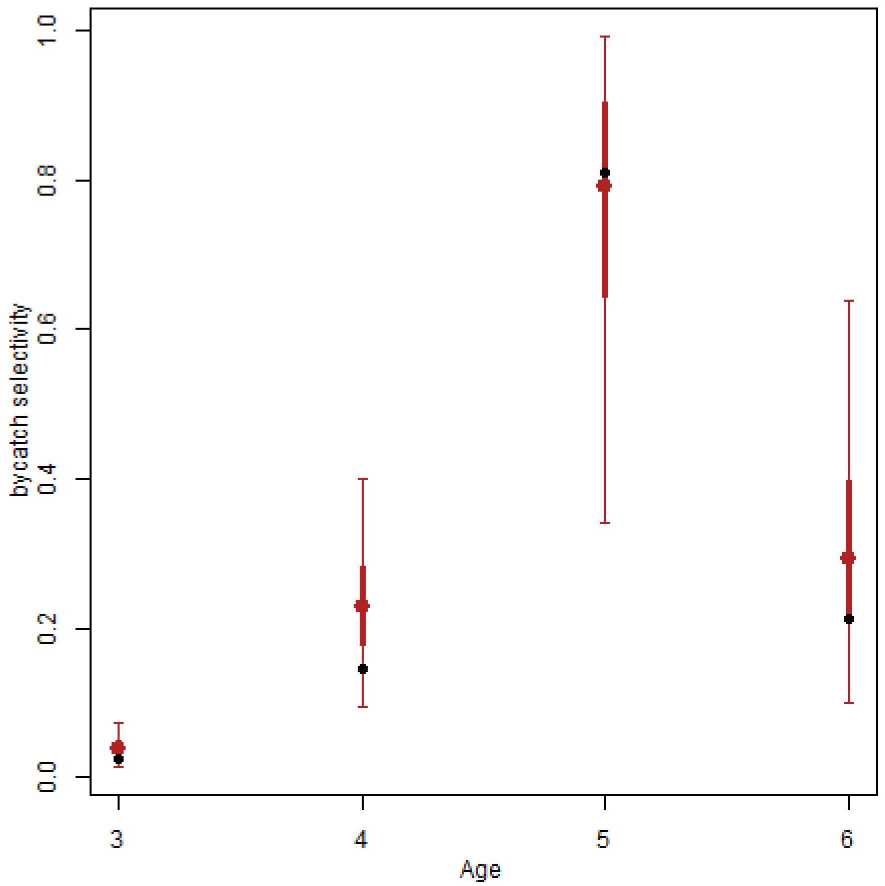

Figure S13. Model estimates of bycatch selectivity-at-age relative to true simulated values. The true bycatch selectivity-at-age values specified in the simulation are shown in black, and model estimates based on fits to fake data simulated (with errors) are shown in red. The median model estimates are indicated by the circles, with 50% and 95% credible intervals indicated by thick and thin lines respectively

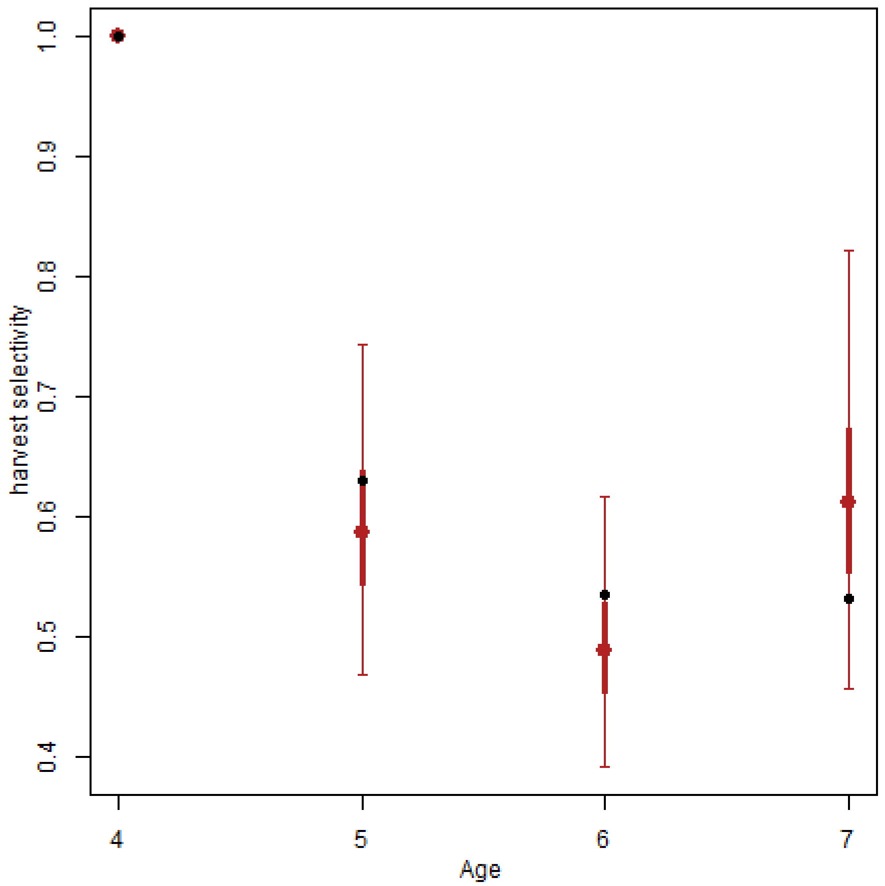

Figure S14. Model estimates of directed fishery selectivity-at-age relative to true simulated values. The true directed fishery selectivity-at-age values specified in the simulation are shown in black, and model estimates based on fits to fake data simulated (with errors) are shown in red. The median model estimates are indicated by the circles, with 50% and 95% credible intervals indicated by thick and thin lines respectively
